# A Single‐Atom Manganese Nanozyme Mn‐N/C Promotes Anti‐Tumor Immune Response via Eliciting Type I Interferon Signaling

**DOI:** 10.1002/advs.202305979

**Published:** 2024-02-02

**Authors:** Wen Qiao, Jingqi Chen, Huayuan Zhou, Cegui Hu, Sumiya Dalangood, Hanjun Li, Dandan Yang, Yu Yang, Jun Gui

**Affiliations:** ^1^ State Key Laboratory of Systems Medicine for Cancer Renji‐Med X Clinical Stem Cell Research Center Ren Ji Hospital School of Medicine Shanghai Jiao Tong University Shanghai 200127 China; ^2^ Institute of Molecular Medicine (IMM) Renji Hospital School of Medicine Shanghai Jiao Tong University Shanghai 200127 China; ^3^ Evergrande Center for Immunologic Diseases Ann Romney Center for Neurologic Diseases Harvard Medical School and Mass General Brigham Boston MA 02115 USA

**Keywords:** anti‐tumor immunity, nanomedicine, ROS, single‐atom nanozyme, type I interferon

## Abstract

Tumor microenvironment (TME)‐induced nanocatalytic therapy is a promising strategy for cancer treatment, but the low catalytic efficiency limits its therapeutic efficacy. Single‐atom catalysts (SACs) are a new type of nanozyme with incredible catalytic efficiency. Here, a single‐atom manganese (Mn)‐N/C nanozyme is constructed. Mn‐N/C catalyzes the conversion of cellular H_2_O_2_ to ∙OH through a Fenton‐like reaction and enables the sufficient generation of reactive oxygen species (ROS), which induces immunogenic cell death (ICD) of tumor cells and significantly promotes CD8^+^T anti‐tumor immunity. Moreover, RNA sequencing analysis reveals that Mn‐N/C treatment activates type I interferon (IFN) signaling, which is critical for Mn‐N/C‐mediated anti‐tumor immune response. Mechanistically, the release of cytosolic DNA and Mn^2+^ triggered by Mn‐N/C collectively activates the cGAS‐STING pathway, subsequently stimulating type I IFN induction. A highly efficient single‐atom nanozyme, Mn‐N/C, which enhances anti‐tumor immune response and exhibits synergistic therapeutic effects when combined with the anti‐PD‐L1 blockade, is proposed.

## Graphical abstract

This study reports a single‐atom manganese (Mn)‐N/C nanozyme with highly efficient peroxidase‐like activity. Mn‐N/C‐mediated catalytic reaction prompts the concurrent release of cytosolic DNA and Mn^2+^, which coordinately activate cGAS‐STING‐IFN signaling cascade, thereby robustly promoting CD8^+^T anti‐tumor immunity and suppressing tumor growth. Mn‐N/C exhibits synergistic effects when combined with anti‐PD‐L1 blockade, offering a promising avenue to improve the efficacy of immunotherapy.

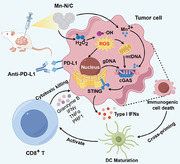

## 1 Introduction

Cancer remains a fatal threat to human health, imposing substantial burdens on both the global economy and life quality.^[^ ^1^ ^]^ Conventional cancer therapies, such as chemotherapy and radiotherapy, directly eradicate tumor cells but concurrently inflict damage to the normal cells.^[^ ^2^ ^]^ Immunotherapy, exemplified by immune checkpoint inhibitors (ICIs) designed to reinvigorate anti‐tumor immune response, has emerged as a promising therapeutic approach across various malignancies. However, due to tumor microenvironmental suppression, the therapeutic efficacy of immunotherapy is limited, with only a low rate of patients exhibiting responsiveness to ICIs.^[^ ^3^ ^]^ How to overcome the immunosuppressive tumor microenvironment (TME) remains a major challenge.

Chemodynamic therapy (CDT) represents a novel therapeutic approach known for its capacity to instigate the generation of oxidizing hydroxyl radical (∙OH) production through Fenton‐like reactions facilitated by nanomaterials.^[^ ^4^ ^]^ Nanozyme, a class of inorganic nanomaterial, is capable of performing catalytic activities similar to natural enzymes. For decades, nanozymes possessing peroxidase‐like activity have demonstrated their great potential for converting hydrogen peroxide (H_2_O_2_) and oxygen (O_2_) into toxic reactive oxygen species (ROS).^[^ ^5^ ^]^ ROS, known as potent oxidants, exert anti‐tumor effects by directly killing tumor cells as well as modulating host immune response.^[^ ^6^ ^]^ The excessive H_2_O_2_ in the TME provides sufficient substrates for potentiating the effects of nanozymes‐mediated CDT,^[^ ^7^ ^]^ whereas H_2_O_2_ deficiency in normal cells mitigates the likelihood of severe Fenton reaction, thereby minimizing damage to normal cells.^[^ ^8^ ^]^ Therefore, designing the nanozyme with highly catalytic efficiency to generate ROS is critical for enhancing the effectiveness of CDT against tumors.

Single‐atom catalysts (SACs), a new type of nanozyme, have been proven to effectively improve catalytic activity owing to their uniform reaction sites and highly unsaturated coordination environment.^[^ ^9^ ^]^ Amplifying the surface‐active sites is considered a super effective approach to accelerate the catalytic speed. SACs maximize their surface area utilization at the atomic level.^[^ ^10^ ^]^ Notably, iron (Fe)‐based SACs display heightened catalytic activity, engendering substantial ROS generation.^[^ ^11^ ^]^ Fe‐SACs have been investigated for catalytic tumor therapy and shown to effectively trigger the tumor‐specific Fenton reaction under an acidic tumor microenvironment, leading to efficient tumor suppression.^[^ ^12^ ^]^ Furthermore, Copper (Cu)‐based SAC demonstrates superior superoxide (SOD)‐like activity, harboring significant potential as therapeutic nanomedicines for sepsis treatment.^[^ ^13^ ^]^ Manganese (Mn), a recognized biologically essential element, assumes a pivotal role as a cofactor in various metalloenzymes and nanozymes, including manganese catalase (MnCAT), manganese superoxide dismutase (MnSOD) and peroxidase (POD).^[^ ^14^ ^]^ Mn‐based nanozymes have been instrumental in inducing a Fenton‐like reaction.^[^ ^15^ ^]^ Nevertheless, the therapeutic potency of Mn‐based SAC against tumors has not been fully explored, and its role in the modulation of tumor immune microenvironment is largely unknown.

In this study, we constructed an Mn‐based SAC (Mn‐N/C) by coordination of single‐atom Mn nano‐catalyst encapsulated in zeolite imidazole frameworks (ZIF‐8). ZIF‐8 exhibits significant advantages in adsorption and drug loading due to its tunable pore channels and large specific surface area.^[^ ^16^ ^]^ We found that Mn‐N/C exhibited pronounced Fenton activity and ROS generation. Intratumoral administration of Mn‐N/C significantly suppressed tumor growth and promoted CD8^+^T anti‐tumor immune response. We further revealed that the cytosolic DNA and Mn^2+^ released by Mn‐N/C coordinately activated the cyclic GMP‐AMP synthase (cGAS)‐stimulator of interferon genes (STING)signaling, consequently stimulating type I interferon (IFN) induction, which was critical for Mn‐N/C‐mediated anti‐tumor immune response. Moreover, Mn‐N/C exhibited synergistic therapeutic effects when combined with anti‐PD‐L1 blockade, suggesting that Mn‐N/C‐mediated CDT could be a new strategy for improving the efficacy of cancer immunotherapy.

## 2 Results

### 2.1 Synthesis and Characterization of Mn‐N/C Single‐Atom Nanozyme

**Figure 1:**
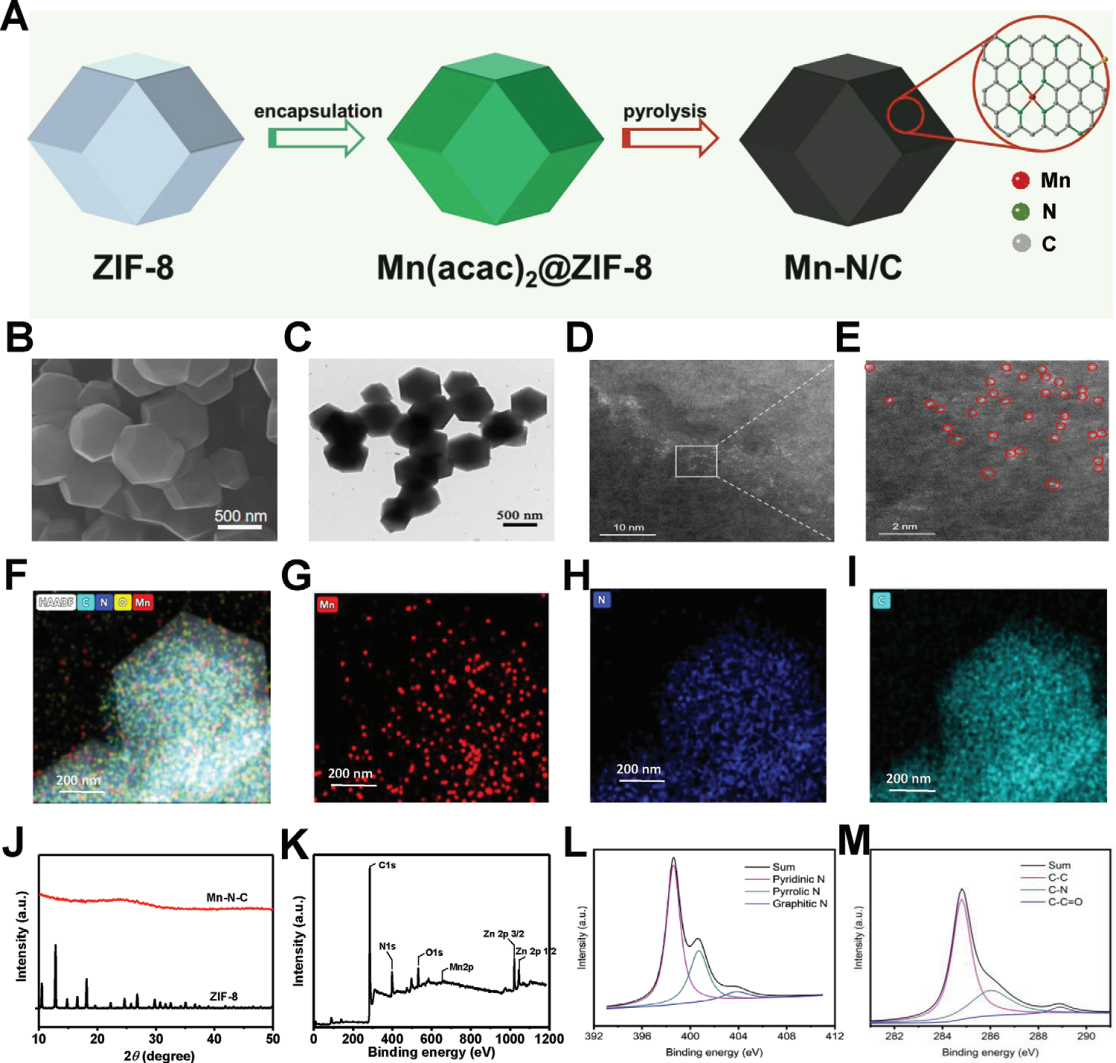
Characterization of single‐atom manganese nanozyme Mn‐N/C. A) Schematic illustration of the formation of Mn‐N/C single‐atom nanozyme. B) SEM micrograph. C) TEM micrograph. D,E) AC‐HAADF‐STEM micrograph. F–I) the EDS mapping of Mn, N, and C of prepared Mn‐N/C. J) XRD patterns of the ZIF‐8 and Mn‐N/C. K) XPS survey spectra of Mn‐N/C. L,M) XPS spectra for C 1s and N 1s of Mn‐N/C.

The synthesis process of Mn‐N/C single‐atom nanozyme was illustrated in **Figure** **1**A, where the manganese precursor, Mn(acac)2, was absorbed in ZIF‐8 prior to pyrolysis. The morphology of the synthesized Mn‐N/C was characterized by scanning electron microscopy (SEM) and transmission electronic microscopy (TEM). The images showed that the size of Mn‐N/C nanozymes ranged from 600 to 700 nm, exhibiting a consistent dodecahedral structure (Figure 1B,C; Figure S1A, Supporting Information). Notably, high‐resolution imaging by aberration‐corrected annular high‐angle dark‐field scanning transmission electron microscopy (AC HAADF‐STEM) revealed the homogeneous dispersion of single Mn atoms within the nanostructure, confirming the successful preparation of single‐atom Mn‐N/C nanozymes (Figure 1D,E). Furthermore, energy dispersive spectrometry (EDS) mapping was also performed to characterize the elemental distribution within single‐atom nanozymes. The results revealed a uniform distribution of manganese (Mn), nitrogen (N), and carbon (C) throughout the prepared Mn‐N/C single‐atom nanozymes (Figure 1F–I), which provided further evidence for the successful synthesis of the single‐atom nanozymes. The structural properties of Mn‐N/C single‐atom nanozyme were characterized by X‐ray diffraction (XRD) and X‐ray photoelectron spectroscopy (XPS). In contrast to the prominent diffraction signal observed in the ZIF‐8, no significant signal corresponding to metal–organic frameworks (MOFs)‐like structure was observed in the Mn‐N/C single‐atom nanozyme (Figure 1J). Meanwhile, the XRD pattern showed a distinct diffraction peak at 25.3°, which could be attributed to the (002) plane of graphitic carbon. These results suggested that the Mn‐N/C single‐atom nanozyme was successfully synthesized, with evident perturbation in the original crystal structure of ZIF‐8 during the process. In addition, the binding states of Mn, C, and N in Mn‐N/C single‐atom nanozyme were analyzed by XPS survey spectra (Figure 1K; Figure S1B, Supporting Information). The presence of pyridinic N, pyrrolic N, and graphitic N in nanozyme was detected by high‐resolution XPS spectra of N 1s analysis (Figure 1L). The high‐resolution analysis of C 1s spectra revealed peaks at 284.8, 285.8, and 288.9 eV, corresponding to C─C, C─N, and C═O, respectively (Figure 1M). Taken together, all these results provide conclusive evidence that the Mn‐N/C single‐atom nanozyme was successfully prepared.

### 2.2 Enzyme‐Mimicking Activity of Mn‐N/C Single‐Atom Nanozyme

**Figure 2:**
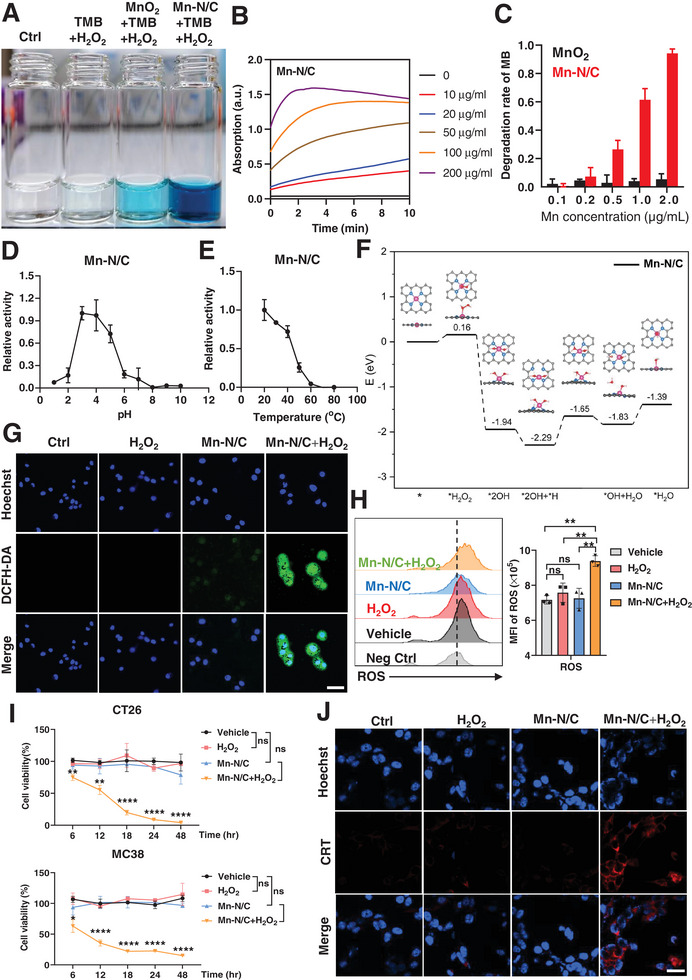
The peroxidase‐like activity of Mn‐N/C and ROS generation. A) Typical photographs of TMB reaction solutions oxidized by Mn‐N/C or MnO_2_ when incubated at room temperature. B) The time‐dependent absorbance changes at 652 nm in the absence (black) or presence of different concentrations of Mn‐N/C. C) ROS scavenging ability of Mn‐N/C and MnO_2_ determined by UV–vis absorbance based on methylene blue (MB). Data are shown as mean±SEM (*n* = 3). D) The peroxidase‐like catalytic activity of Mn‐N/C under different pH conditions. Data are shown as mean±SEM (*n* = 3). E) The peroxidase‐like catalytic activity of Mn‐N/C under different temperatures. Data are shown as mean±SEM (*n* = 3). F) DFT studies on the peroxidase‐like activity of Mn‐N/C. In the energy profiles, the most favorable paths of H_2_O_2_ dissociation into surface OH species in the acidic condition. The Mn, C, N, and H atoms were given in pink, gray, blue, and white, respectively. G) Confocal imaging of ROS generation in CT26 tumor cells after treatment with H_2_O_2_ or Mn‐N/C (100 µg/mL). DCFH‐DA was used as a fluorescent probe for ROS imaging. Scale bar: 50 µm. H) FACS analysis (left) and the quantification (right) of ROS production in MC38 tumor cells after vehicle or Mn‐N/C treatment for 24 h. Quantitative data are shown as mean±SEM (*n* = 3). One‐way ANOVA and Sidak's multiple comparisons test were performed. I) The cell viability of CT26 and MC38 tumor cells treated with H_2_O_2_ or Mn‐N/C for the indicated time points by CCK8 assays. Quantitative data are shown as mean±SEM (*n* = 3). Two‐way ANOVA (mixed model) and Sidak's multiple comparisons test were performed. J) Immunofluorescence staining of CRT in MC38 tumor cells after treatment with H_2_O_2_ or Mn‐C/N for 24 h. Scale bar, 20 µm. ^*^, *p* < 0.05; ^**^, *p* < 0.01; ^***^, *p* < 0.001; ^****^, *p* < 0.0001; ns, no significance

We investigated the catalytic oxidation ability of the obtained Mn‐N/C single‐atom nanozyme by using 3,3′,5,5′‐tetramethylbenzidine (TMB) as a chromogenic substrate. Upon the addition of Mn‐N/C, the oxidation of colorless TMB by Mn‐N/C generated a blue oxide (**Figure** **2**A). The production of TMB oxide was quantified by measuring the absorbance at 652 nm. We found that the absorbance at 652 nm was increased along with time, and the absorbance was positively correlated with the concentrations of Mn‐N/C (Figure 2B). We further measured the dynamic change of absorbance at different wavelengths in the presence of different concentrations of Mn‐N/C (Figure S2A, Supporting Information), confirming the oxidase‐like activity of Mn‐N/C. Subsequent detection of reactive oxygen species (ROS) using Methylene Blue (MB) as a free radical indicator showed a concentration‐dependent degradation rate of MB upon the addition of Mn‐N/C, indicating ROS generation (Figure 2C). Notably, Mn‐N/C exhibited significantly higher catalytic activity when compared to MnO_2_ (Figure 2A,C; Figure S2B,C, Supporting Information). Moreover, electron paramagnetic resonance (EPR) analysis revealed that Mn‐N/C effectively catalyzed the conversion of H_2_O_2_ to ∙OH (Figure S2D, Supporting Information). In addition, we noticed that Mn‐N/C demonstrated peroxidase (POD)‐like catalytic activity at acidic pH conditions, but did not exhibit such catalytic activity at alkaline pH conditions (Figure 2D), emphasizing its potential in the acidic tumor microenvironment. We also examined the catalytic activity of Mn N/C across different temperatures and found that Mn‐N/C displayed significant catalytic activity across a broad temperature range (20–60 °C) (Figure 2E).

To gain deeper insights into the potential catalytic mechanism of Mn‐N/C, we performed density functional theory (DFT) calculations. The energy profile diagram unveiled that H_2_O_2_ exhibited weak adsorption energy with barriers of 0.16 eV on the Mn‐N/C surface, where the bond length of Mn─O was calculated to be 2.50 Å. Thus, the adsorbed H_2_O_2_ would be easily dissociated into two OH species with reaction energies of −2.10 eV, indicating the strong ability to generate ∙OH. Interestingly, the two OH species were simultaneously adsorbed on the Mn atom. Subsequent analysis revealed strong adsorption of the H atom on Mn‐N/C, positioning the atomic H at the top sites of atomic N. Starting with 2OH+H, one of the OH species reacted with the H atom to form an H_2_O molecule with barriers of 0.64 eV. Further assessment of the desorption energy of the H_2_O molecule indicated a value of −0.18 eV, implying the facile removal of the H_2_O molecule. These calculations suggest that the OH species reacting with the H atom is the rate‐determining step in the peroxidase‐like catalysis. The low reaction energy (0.64 eV) distinctly underscores the high POD‐like catalytic activity of Mn‐N/C (Figure 2F).

To evaluate the cellular generation of reactive oxygen species (ROS), we used 2,7‐dichlorodi‐hydrofluorescein diacetate (DCFH‐DA) as a fluorescent probe for ROS imaging in CT26 colon carcinoma cells. Upon ROS oxidation, DCFH‐DA is converted to DCFH, emitting green fluorescence. To mimic the physiochemical condition in tumors, 100 µm H_2_O_2_ was added to the treatment. Confocal imaging revealed that only Mn‐N/C plus H_2_O_2_ treatment resulted in a substantial ROS generation in CT26 tumor cells, indicating the potent catalytic ability of Mn‐N/C in cells utilizing H_2_O_2_ as a substrate (Figure 2G). Validation of ROS levels using flow cytometry consistently indicated a significant elevation in ROS levels within MC38 colon carcinoma cells upon treatment with Mn‐N/C and H_2_O_2_ (Figure 2H), confirming the Fenton‐like reactions facilitated by Mn‐N/C. Additionally, treatment with Mn‐N/C and H_2_O_2_ notably reduced GSH levels in MC38 tumor cells (Figure S2E, Supporting Information), corresponding with the elevation of ROS levels.

We then examined the cell viability by using a CKK8 assay. The results showed that Mn‐N/C or H_2_O_2_ alone had a negligible effect on tumor cell viability, whereas the combined treatment of Mn‐N/C and H_2_O_2_ remarkably decreased the viability of both CT26 and MC38 tumor cells over time (Figure 2I), indicating the potent cytotoxic effects of Mn‐N/C in tumor cells in the presence of H_2_O_2_. To determine the potential induction of immunogenic cell death (ICD) in tumor cells following Mn‐N/C treatment, we analyzed the expression of calreticulin (CRT), a classical ICD marker. Notably, CRT was evidently induced after Mn‐N/C plus H_2_O_2_ treatment, but not expressed in Mn‐N/C alone or H_2_O_2_‐treated cells (Figure 2J). All these data suggested that Mn‐N/C efficiently produced cellular ROS and induced ICD of tumor cells.

### 2.3 Mn‐N/C Inhibits Tumor Growth and Promotes Anti‐Tumor Immunity

**Figure 3:**
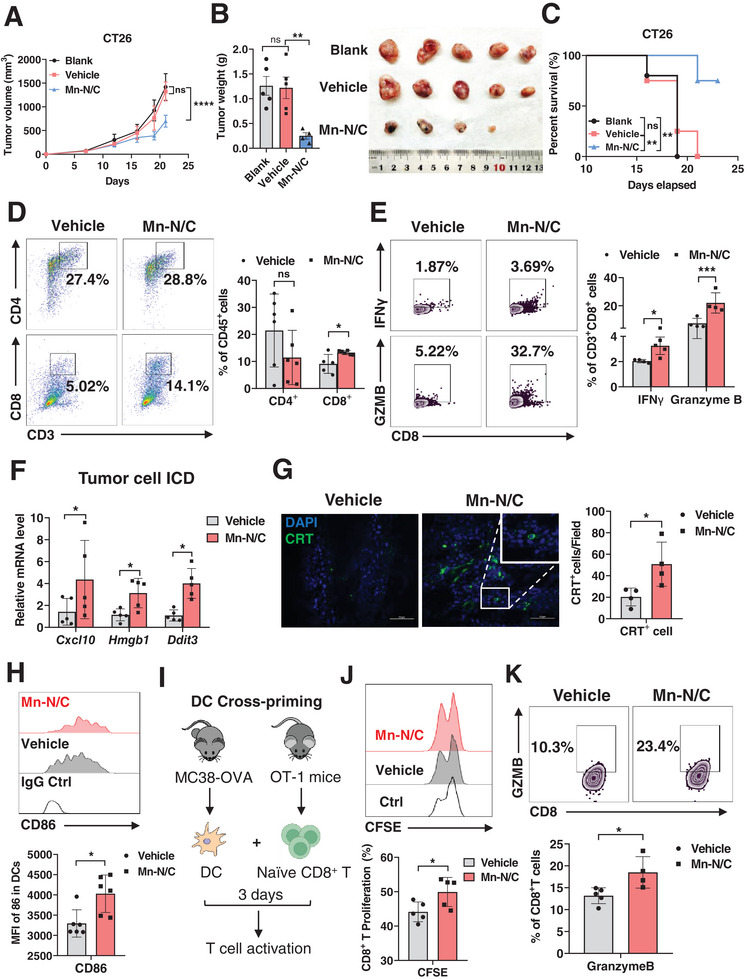
Mn‐N/C inhibits tumor growth and promotes anti‐tumor immunity. A) Tumor growth curve of CT26 in the WT mice intratumorally injected with vehicle, Mn‐N/C, or without treatment (Blank). Data are shown as mean±SEM (*n* = 4–5 mice per group). Two‐way ANOVA (mixed model) and Sidak's multiple comparisons test were performed. B) The images of the tumor mass and tumor weight from CT26 tumor‐bearing mice in blank, vehicle, and Mn‐N/C groups. Data are shown as mean±SEM (*n* = 4–5 mice per group). Two‐tailed unpaired *t*‐test was performed for the comparison between the two groups. C) Kaplan–Meier analysis of mice survival in blank, vehicle, and Mn‐N/C groups as A described (*n* = 4–5 mice per group). D) FACS analysis (left) and quantification (right) of tumor‐infiltrating CD4^+^ and CD8^+^ T cells in the mice treated with vehicle or Mn‐N/C. Data are shown as mean±SEM (*n* = 5–6 mice per group). Two‐way ANOVA and Sidak's multiple comparisons test were performed. E) FACS analysis (left) and quantification (right) of IFNγ^+^ and Granzyme B^+^ of tumor‐infiltrating CD8^+^ T cells in the mice treated with vehicle or Mn‐N/C. Data are shown as mean±SEM (*n* = 4–5 mice per group). Two‐way ANOVA and Sidak's multiple comparisons test were performed. F) Real‐time PCR analysis of *Cxcl10*, *Hmgb1*, *and Ddit3* gene expression levels in the tumor cells (RFP^+^) sorted from MC38‐OVA‐RFP tumor‐bearing mice with vehicle or Mn‐N/C treatment. Data are shown as mean±SEM (*n* = 5). Two‐way ANOVA and Sidak's multiple comparisons test were performed. G) The immunofluorescence (left) and the quantification (right) of CRT in MC38 tumors treated with vehicle or Mn‐N/C. Scale bar, 50 µm. Two‐tailed unpaired *t*‐test was performed for the comparisons between the two groups. H) FACS analysis (above) and quantification (below) of CD86 expression of DCs in the draining lymph nodes from MC38‐OVA‐RFP tumor‐bearing mice treated with vehicle or Mn‐N/C. Data are shown as mean±SEM (*n* = 6 mice per group). Two‐tailed unpaired *t*‐test was performed for the comparisons between the two groups. I) Schematic illustration of the analysis of DC‐cross priming. naïve CD8^+^ T cells purified from OT‐1 mice were co‐cultured with DCs sorted from the draining lymph nodes of MC38‐OVA tumor‐bearing mice with vehicle or Mn‐N/C treatment for 3 days to evaluate T cell activation. J) FACS analysis (above) of CFSE and quantification (below) of T cell proliferation after co‐culturing with DCs for 3 days as I described. Data are shown as mean±SEM (*n* = 4–5 mice per group). Two‐tailed unpaired *t*‐test was performed for the comparisons between the two groups. K) FACS analysis (above) and quantification (below) of Granzyme B^+^ of CD8^+^T cells after co‐culturing with DCs for 3 days as I described. Data are shown as mean±SEM (*n* = 4–5 mice per group). Two‐tailed unpaired *t*‐test was performed for the comparisons between the two groups. ^*^, *p* < 0.05; ^**^, *p* < 0.01; ^***^, *p* < 0.001; ^****^, *p* < 0.0001; ns, no significance

Encouraged by the cytotoxic effect of Mn‐N/C at a cellular level, we further assessed its anti‐tumor therapeutic efficacy in CT26 transplanted tumor models. When the tumor size was ≈60 mm^3^, Mn‐N/C solution dissolved in saline (70 mg kg^−1^, 50 µL) was intratumorally injected into the mice every 3 days. We found that Mn‐N/C administration significantly suppressed CT26 tumor growth, reduced the tumor weight, and prolonged mice survival when compared to the untreated (blank group) and saline (vehicle)‐treated mice (**Figure** **3**A–C). As expected, Mn‐N/C exhibited no discernible impact on the cell viability of normal intestinal epithelial cells (IECs) (Figure S3A, Supporting Information). Accordingly, Mn‐N/C administration did not induce any change in the body weight of mice (Figure S3B, Supporting Information). Additionally, histological assessments via H&E staining of vital organs, including heart, liver, spleen, lung, and kidney, revealed that Mn‐N/C treatment did not cause tissue damage (Figure S3C, Supporting Information), affirming the biological safety of Mn‐N/C for in vivo treatment.

Next, we sought to assess the immune response in the TME upon Mn‐N/C administration. As T cells are the key players in TME,^[^ ^17^ ^]^ we analyzed the T cell infiltration in tumor tissues and observed that the infiltration of CD8^+^T cells was notably increased in Mn‐N/C‐treated tumors when compared to the control tumors (Figure 3D). The infiltration of CD4^+^T cells showed no difference. Furthermore, the cytotoxic function of CD8^+^T cells was enhanced in Mn‐N/C‐treated tumors, as evidenced by elevated production of cytotoxic molecules IFNγ and Granzyme B in the tumor‐infiltrating CD8^+^T cells after Mn‐N/C treatment (Figure 3E). Similarly, in another transplanted colon cancer model established by ovalbumin (OVA)‐expressing MC38 (MC38‐OVA) tumor cells, the intratumoral administration of Mn‐N/C significantly blunted tumor growth (Figure S3D, Supporting Information), augmented CD8^+^T cell infiltration (Figure S3E, Supporting Information) and enhanced the cytotoxic function of CD8^+^ T cells (Figure S3F, Supporting Information). All these data suggested that in vivo treatment with Mn‐N/C promoted CD8^+^ T anti‐tumor immune response.

ICD has been known to initiate CD8^+^T cell‐mediated adaptive immune response, facilitated by the releases of damage‐associated molecular patterns (DAMPs). These DAMPs encompass cell surface exposure of CRT, heat‐shock proteins (HSP70 and HSP90), and high‐mobility group box‐1 (HMGB1).^[^ ^18^ ^]^ To determine whether Mn‐N/C treatment triggers ICD of tumor cells in vivo, we sorted out the tumor cells (RFP^+^) from MC38 tumor‐bearing mice and analyzed the expression of ICD markers including *Cxcl10*, *Hmgb1*, and *Ddit3*. The results showed that mRNA levels of these ICD markers were significantly increased in tumor cells from Mn‐N/C‐treated tumors when compared to those from vehicle‐treated tumors (Figure 3F). Moreover, immunofluorescence staining of CRT within the tumors exhibited a substantial induction after Mn‐N/C treatment (Figure 3G), signifying the induction of ICD in tumor cells by Mn‐N/C treatment in vivo. In addition to DAMPs, ICD also leads to the release of tumor‐associated antigens, which promote dendritic cell (DC) maturation, enhancing cross‐priming and subsequent activation of CD8^+^T cells.^[^ ^19^ ^]^ Indeed, we found that DCs from draining lymph nodes of Mn‐N/C‐treated mice exhibited higher expression of the DC maturation marker CD86 (Figure 3H). To analyze DC cross‐priming, we sorted out DCs (CD11c^+^CD103^+^MHCII^high^) from draining lymph nodes of MC38‐OVA tumor‐bearing mice subjected to Mn‐N/C or vehicle treatment and co‐cultured them with OT‐1 CD8^+^T cells, known to react with the OVA peptide 257–264, for 3 days (Figure 3I). Remarkably, DCs from Mn‐N/C‐treated mice were more potent in stimulating CD8^+^T cell proliferation and Granzyme B production (Figure 3J,K), suggesting that Mn‐N/C treatment augments the cross‐priming capacity of DCs.

Collectively, all these data suggest that Mn‐N/C fosters CD8^+^ T anti‐tumor immunity by inducing ICD in tumor cells.

### 2.4 Mn‐N/C Activates Type I IFN Signaling by cGAS/STING‐Dependent Cytosolic DNA Sensing

**Figure 4:**
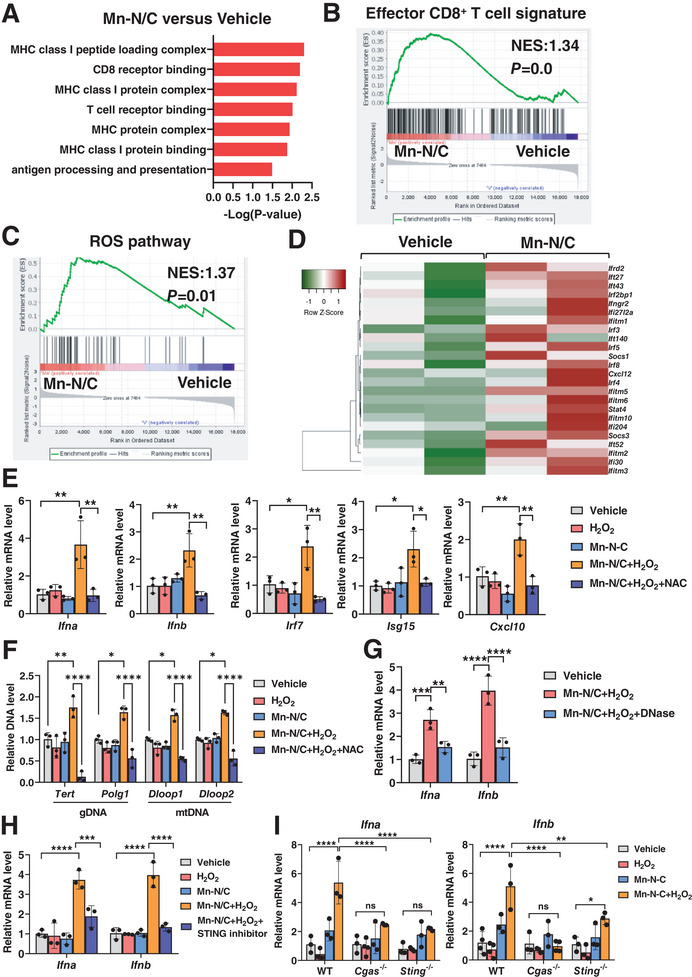
Mn‐N/C activates type I IFN signaling by cGAS/STING‐dependent cytosolic DNA sensing. A) GO enrichment gene analysis of pathways in tumor tissues of MC38‐bearing mice with vehicle or Mn‐N/C treatment. B) GSEA analysis of enrichment of CD8^+^ T cell effector signatures in tumor tissues of MC38 tumor‐bearing mice with vehicle or Mn‐N/C treatment. C) GSEA analysis of enrichment of ROS pathway signatures in tumor tissues of MC38‐bearing mice with vehicle or Mn‐N/C treatment. D) Heatmap of type I IFN gene signatures in tumor tissues of MC38‐bearing mice with vehicle or Mn‐N/C treatment. E) Real‐time PCR analysis of *Ifna*, *Ifnb*, *Irf7*, *Isg15*, *and Cxcl10* gene expression levels in MC38 tumor cells treated with H_2_O_2_, Mn‐N/C, or plus with NAC (1 mm) for 24 h. Data are shown as mean±SEM (*n* = 3). Two‐tailed unpaired *t*‐test was performed for the comparisons between the two groups. F) Cytosolic amount of gDNA and mtDNA in MC38 tumor cells treated with H_2_O_2_, Mn‐N/C, or plus with NAC (1 mm) treatment for 24 h. Data are shown as mean±SEM (*n* = 3). Two‐way ANOVA and Sidak's multiple comparisons test were performed. G) Real‐time PCR analysis of *Ifna* and *Ifnb* expression in MC38 tumor cells treated with Mn‐N/C and H_2_O_2_, or plus with DNase for 24 h. Data are shown as mean±SEM (*n* = 3). Two‐way ANOVA and Sidak's multiple comparisons test were performed. H) Real‐time PCR analysis of *Ifna* and *Ifnb* expression in MC38 tumor cells treated with Mn‐N/C, H_2_O_2_, or plus with STING inhibitor H151 for 24 h. Data are shown as mean±SEM (*n* = 3). Two‐way ANOVA and Sidak's multiple comparisons test were performed. I) Real‐time PCR analysis of *Ifna* and *Ifnb* expression in WT, *Cgas* ^−/−^, *Sting* ^−/−^ MC38 tumor cells treated with H_2_O_2_, Mn‐N/C for 24 h. Data are shown as mean±SEM (*n* = 3). Two‐way ANOVA and Sidak's multiple comparisons test were performed. ^*^, *p* < 0.05; ^**^, *p* < 0.01; ^***^, *p* < 0.001; ^****^, *p* < 0.0001; ns, no significance

To further elucidate the mechanism underlying the enhanced immune response by Mn‐N/C treatment, we conducted RNA sequencing of MC38 tumors with Mn‐N/C or vehicle treatment. Through functional enrichment analysis, we observed a significant upregulation in the enrichment for antigen presentation and CD8^+^T cell activation pathways in Mn‐N/C‐treated tumors (**Figure** **4**A). Employing Gene Set Enrichment Analysis (GSEA) to evaluate T cell effector signatures, we noted heightened expression of T cell cytotoxic functional markers in Mn‐N/C‐treated tumors (Figure 4B). Considering the substantial presence of H_2_O_2_ within tumors (Figure S4A, Supporting Information) and the reduction in GSH levels following Mn‐N/C treatment (Figure S4B, Supporting Information), we found a predominant increase in gene signatures linked to ROS pathway in Mn‐N/C‐treated tumors (Figure 4C). Interestingly, among the differentially expressed genes, we observed robust upregulation in the transcripts of type I IFN‐stimulated genes (ISGs) in Mn‐N/C‐treated tumors when compared to the control tumors (Figure 4D), indicating that type I IFN signaling was activated in the tumors after Mn‐N/C treatment. Guided by the RNA‐seq data, we further confirmed IFNα/β and ISGs expression in tumor cells. The results revealed that Mn‐N/C plus H_2_O_2_ treatment notably stimulated high expression levels of IFNα, IFNβ, and ISGs including *Irf7*, *Isg15*, and *Cxcl10* in MC38 tumor cells. Conversely, the use of N‐acetylcysteine (NAC), a ROS scavenger, significantly reduced the expression of IFNα/β and ISGs (Figure 4E), indicating that the activated type I IFN signaling was dependent on ROS production mediated by Mn‐N/C.

Type I IFNs, primarily IFNα/β, can be induced following the recognition of cytosolic DNA by the cGAS‐STING pathway.^[^ ^20^ ^]^ In fact, we observed a high abundance of both mitochondrial DNA (mtDNA) and genomic DNA (gDNA) in the cytosol upon Mn‐N/C plus H_2_O_2_ treatment, whereas NAC robustly eliminated the accumulation of cytosolic mtDNA and gDNA (Figure 4F). Importantly, DNase treatment significantly reduced the production of type I IFNs, suggesting that DNA is required for Mn‐N/C‐mediated induction of type I IFNs (Figure 4G). To identify whether the cGAS‐STING pathway may contribute to Mn‐N/C‐induced type I IFNs production, we pre‐treated MC38 tumor cells with a STING inhibitor (H151) and observed a significant suppression type I IFNs induction upon Mn‐N/C plus H_2_O_2_ treatment (Figure 4H). Accordingly, cGAS and STING deficiency in MC38 tumor cells resulted in the loss of type I IFNs induction upon Mn‐N/C plus H_2_O_2_ treatment (Figure 4I). All these data suggest that Mn‐N/C activates type I IFN signaling through cGAS/STING‐dependent cytosolic DNA sensing.

Of note, Mn^2+^ itself acts as a potent cGAS‐STING activator, significantly enhancing CD8^+^T anti‐tumor immunity.^[^ ^21^ ^]^ Employing inductively coupled plasma mass spectrometry (ICP‐MS), we detected the presence of Mn^2+^ (≈20 nm) in the cell culture supernatant upon Mn‐N/C plus H_2_O_2_ treatment (Figure S4C, Supporting Information). Indeed, 20 nm Mn^2+^ induced a modest elevation of IFNα/β expression. Apparently, Mn‐N/C plus H_2_O_2_ treatment exhibited a greater IFNα/β induction (Figure S4D, Supporting Information), implying a potential coordinated activation of cGAS‐STING signaling by cytosolic DNA and Mn^2+^ released upon Mn‐N/C treatment. Thus, we assume that Mn‐N/C may have advantages over Mn^2+^. To compare the therapeutic efficacy of Mn‐N/C and MnCl_2_, we intratumorally injected MnCl_2_ (at 5 mg kg^−1^ as previously reported) and Mn‐N/C into CT26 tumor‐bearing mice. Remarkably, Mn‐N/C administration demonstrated superior tumor suppression (Figure S4E, Supporting Information) and prolonged mice survival compared to MnCl_2_‐treated mice (Figure S4F, Supporting Information). Moreover, we observed a notable increase in the frequency of CD8^+^T cells and their enhanced cytotoxic activity in mice treated with Mn‐N/C compared to those receiving MnCl_2_ (Figure S4G,H, Supporting Information), suggesting a more robust anti‐tumor immune response elicited by Mn‐N/C.

### 2.5 Type I Interferon Signaling Is Critical for Mn‐N/C‐Mediated Anti‐Tumor Immune Response

**Figure 5:**
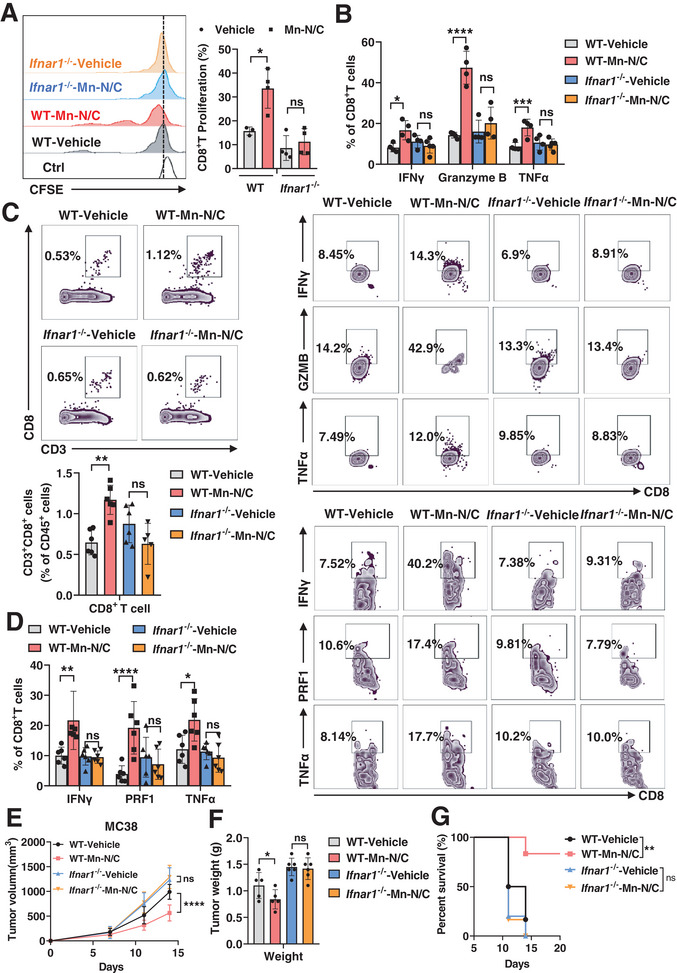
Type I interferon signaling is critical for Mn‐N/C‐mediated anti‐tumor immune response. A) FACS analysis (left) of CFSE and the quantification (right) of OT‐1 T cell proliferation after co‐cultured with DCs sorted from lymph nodes of WT and *Ifnar1^−/−^* MC38‐OVA tumor‐bearing mice with vehicle or Mn‐N/C treatment. Data are shown as mean±SEM (*n* = 4 per group). Two‐way ANOVA and Sidak's multiple comparisons test were performed. B) FACS analysis (below) and the quantification (above) of IFNγ^+^, Granzyme B^+^, TNFα^+^ of OT‐1 CD8^+^ T cells after co‐culture with DCs as A described. Data are shown as mean±SEM (*n* = 4 mice per group). Two‐way ANOVA and Sidak's multiple comparisons test were performed. C) FACS analysis (above) and the quantification (below) of tumor‐infiltrating CD8^+^ T cells in WT and *Ifnar1^−/−^* MC38 tumor‐bearing mice with vehicle or Mn‐N/C treatment. Data are shown as mean±SEM (*n* = 5–6 mice per group). Two‐way ANOVA and Sidak's multiple comparisons test were performed. D) FACS analysis (right) and the quantification (left) of IFNγ^+^, PRF1^+^ (Perforin), TNFα^+^ of CD8^+^ T cells in WT and *Ifnar1^−/−^* MC38 tumor‐bearing mice with vehicle or Mn‐N/C treatment. Data are shown as mean±SEM (*n* = 5–6 mice per group). Two‐way ANOVA and Sidak's multiple comparisons test were performed. E) Tumor growth curve of MC38 in WT and *Ifnar1^−/−^* mice with vehicle or Mn‐N/C treatment. Data are shown as mean±SEM (*n* = 5–6 mice per group). Two‐way ANOVA (mixed model) and Sidak's multiple comparisons test were performed. F) MC38 tumor weight in WT and *Ifnar1^−/−^* mice with vehicle or Mn‐N/C treatment 14 days after tumor cell inoculation. Data are shown as mean±SEM (*n* = 5–6 mice per group). G) Kaplan–Meier analysis of WT and *Ifnar1* ^−/−^ mice survival with vehicle or Mn‐N/C treatment (*n* = 5–6 mice per group). ^*^, *p* < 0.05; ^**^, *p* < 0.01; ^***^, *p* < 0.001; ^****^, *p* < 0.0001; ns, no significance

Type I IFNs elicit the downstream signaling via binding the surface Interferon Alpha Receptor 1 (IFNAR1) to stimulate the transcription of numerous ISGs.^[^ ^22^ ^]^ To investigate whether type I IFN signaling was critical for Mn‐N/C‐activated immune response, we inoculated MC38‐OVA tumor cells into WT and *Ifnar1^−/−^* mice followed by Mn‐N/C or vehicle administration. We sorted out DCs from the draining lymph nodes of these tumor‐bearing mice and co‐cultured them with OT‐1 CD8^+^T cells to assess DC cross‐priming by analyzing CD8^+^T cell proliferation and cytotoxic function. The results showed that the enhanced DC cross‐priming capacity by Mn‐N/C treatment was diminished in *Ifnar1* ^−/−^ mice (**Figure** **5**A,B). We further determined the CD8^+^T cell immune response in MC38 tumor‐bearing WT and *Ifnar1* ^−/−^ mice after Mn‐N/C administration. Likewise, the improved effects of Mn‐N/C treatment on the infiltration and cytotoxic activity of CD8^+^T cells inside MC38 tumors were eliminated in *Ifnar1* ^−/−^ mice (Figure 5C,D). Importantly, the therapeutic efficacy of Mn‐N/C against tumor growth was absent in *Ifnar1* ^−/−^ mice (Figure 5E–G). All these data suggested type I IFN signaling is critical for Mn‐N/C‐mediated anti‐tumor immune response.

### 2.6 Combination of Mn‐N/C and PD‐L1 Blockade Synergistically Suppresses Tumor Growth

**Figure 6:**
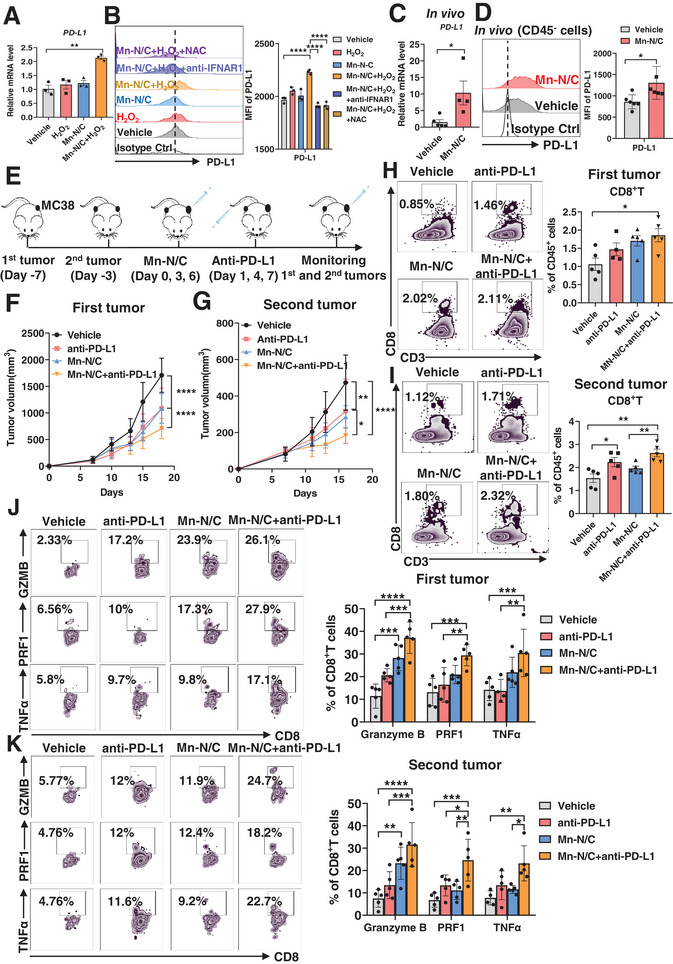
A combination of Mn‐N/C and PD‐L1 blockade synergistically suppresses tumor growth. A) Real‐time PCR analysis of PD‐L1 mRNA expression of MC38 tumor cells treated with H_2_O_2_, Mn‐N/C for 24 h. Quantitative data are shown as mean±SEM (*n* = 3). One‐way ANOVA and Sidak's multiple comparisons test were performed. B) FACS analysis (left) and the quantification (right) of PD‐L1 levels on the surface of MC38 tumor cells treated with H_2_O_2_, Mn‐N/C, or plus with anti‐IFNAR1 antibody or NAC treatment for 24 h. Quantitative data are shown as mean±SEM (*n* = 3). One‐way ANOVA and Sidak's multiple comparisons test were performed. C) Real‐time PCR analysis of PD‐L1 mRNA expression of tumor cells (RFP^+^) sorted from MC38‐OVA tumor‐bearing mice with vehicle or Mn‐N/C treatment. Quantitative data are shown as mean±SEM (*n* = 3). Two‐tailed unpaired *t*‐test was performed for the comparisons between the two groups. D) FACS analysis (left) and the quantification (right) of PD‐L1 levels in CD45^−^ cells from MC38 tumor‐bearing mice with vehicle or Mn‐N/C treatment. Data are shown as mean±SEM (*n* = 6 mice per group). Two‐tailed unpaired *t*‐test was performed for the comparisons between the two groups. E) Schematic illustration of a bilateral model of MC38 tumors subcutaneously implanted on C57BL/6 mice and treated with vehicle or Mn‐N/C, or plus with anti‐PD‐L1 antibody. F,G) Tumor growth curve of the first tumor (F) and the second tumor (G) of MC38 in the WT mice treated as E described. Data are shown as mean±SEM (*n* = 5 mice per group). Two‐way ANOVA (mixed model) and Sidak's multiple comparisons test were performed. H,I) FACS analysis (left) and the quantification (right) of tumor‐infiltrating CD8^+^ T cells in the first tumor (H) and the second tumor (I) of MC38 tumor‐bearing mice treated as E described. Data are shown as mean±SEM (*n* = 5 mice per group). Two‐tailed unpaired *t*‐test was performed for the comparisons between the two groups. J,K) FACS analysis (left) and the quantification (right) of Granzyme B ^+^, PRF1^+^, TNFα^+^ of CD8^+^ T cells in the first tumor (J) and the second tumor (K) of MC38 tumor‐bearing mice treated as E described. Data are shown as mean±SEM (*n* = 5 mice per group). Two‐way ANOVA and Sidak's multiple comparisons test were performed. ^*^, *p* < 0.05; ^**^, *p* < 0.01; ^***^, *p* < 0.001; ^****^, *p* < 0.0001; ns, no significance

Type I IFN signaling is shown to be positively correlated with the expression of PD‐L1 checkpoint molecule.^[^ ^23^ ^]^ Indeed, we found that the mRNA expression of PD‐L1 was significantly induced in MC38 tumor cells upon Mn‐N/C plus H_2_O_2_ treatment (**Figure** **6**A). Accordingly, the protein level of PD‐L1 was upregulated following Mn‐N/C plus H_2_O_2_ treatment. Mn‐N/C or H_2_O_2_ alone had no effect on PD‐L1 expression (Figure 6B). However, anti‐IFNAR1 or NAC treatment attenuated the increased expression of PD‐L1, indicating that Mn‐N/C induces PD‐L1 expression through the activation of type I IFN signaling. Moreover, in vivo Mn‐N/C treatment significantly upregulated PD‐L1 expression in tumor cells at both mRNA and protein levels (Figure 6C,D).

PD‐L1 positive tumors are more vulnerable to ICIs.^[^ ^24^ ^]^ We surmise that Mn‐N/C treatment may potentiate the sensitization to PD‐L1 blockade. To test whether the combination of Mn‐N/C and anti‐PD‐L1 could amplify the therapeutic efficacy against tumors, we established a bilateral tumor model by subcutaneously inoculating MC38 tumors on both the left and right flanks of each mouse (Figure 6E). The left and right tumors were designated as the primary and distant tumors respectively. When the tumor size reached ≈60 mm^3^, Mn‐N/C solution was intratumorally injected into the primary tumor at days 0, 3, and 6. Anti‐PD‐L1 was intravenously injected on days 1, 4, and 7. The bilateral tumor sizes were monitored. Notably, Mn‐N/C combined with anti‐PD‐L1 displayed a better therapeutic efficacy in primary tumors in comparison to either Mn‐N/C injection alone or anti‐PD‐L1 monotherapy (Figure 6F). This synergistic efficacy was also evident in the distant tumor (Figure 6G). We further evaluate the immune responses in bilateral MC38 tumors post treatments. We observed a notable increase in the infiltration of CD8^+^T cells in both primary and distant tumors within the combination treatment group (Figure 6H,I). Furthermore, the combination treatment significantly augmented the production of cytotoxic molecules, including Granzyme B, Perforin, and TNFα, in both primary and distant tumor‐infiltrating CD8^+^T cells (Figure 6J,K). All these data demonstrate that the combination of Mn‐N/C and PD‐L1 blockade potentiates CD8^+^T anti‐tumor immune response and synergistically suppresses tumor growth.

## 3 Discussion

**Figure 7:**
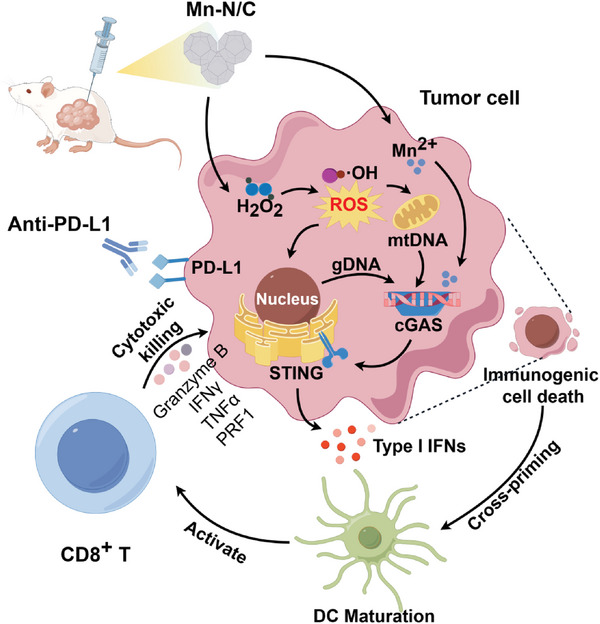
Schematic diagram of Mn‐N/C for activating anti‐tumor immune response. Conceptual and effective mechanisms of Mn‐N/C in triggering immunogenic cell death and remodeling tumor immune microenvironment.

Cancer immunotherapy has recently been demonstrated to be effective and important for tumor treatment.^[^ ^25^ ^]^ However, the presence of “cold” tumors, which have limited response to immunotherapy due to immunological ignorance or the interception of T cell activation, results in the low response of immune checkpoint inhibitors (ICIs) in clinical applications.^[^ ^3^, ^26^ ^]^ To enhance the efficacy of ICIs, various treatments are developing to combine with ICIs for potentiating their responses. Tailored to the specific TME that is characterized by mild acidity, H_2_O_2_ overproduction, low catalase activity, and hypoxia,^[^ ^7^, ^27^ ^]^ chemodynamic therapy (CDT) is an emerging therapeutic strategy by catalyzing H_2_O_2_ through Fenton‐like reactions to generate ROS at tumor sites.^[^ ^28^ ^]^ With the rapid development of Fenton and Fenton‐like nanomaterial, CDT has attracted tremendous attention. The Fenton‐like reaction‐mediated CDT strategies are based on some metal elements like iron, copper, manganese, cobalt, et al.^[^ ^4a^ ^]^ In this study we constructed a new promising single‐atom nanozyme Mn‐N/C with extraordinary catalytic activity. Mn‐N/C in situ administration exerts efficient anti‐tumor effects. Importantly, Mn‐N/C enhances anti‐tumor immune response and exhibits synergistic therapeutic effects when combined with anti‐PD‐L1 blockade, suggesting that Mn‐N/C‐mediated CDT could be a novel strategy for improving cancer immunotherapy (**Figure** **7**).

Single‐atom catalysts (SACs) can stabilize individual metal atoms dispersed on a carrier and demonstrate higher catalytic activity and efficiency due to their high atomic utilization and controllable single‐active sites compared to conventional catalysts.^[^ ^29^ ^]^ Therefore, the development of SACs stands as a potent means to initiate biochemical reactions. Manganese, a trace element, is a key cofactor of numerous metalloenzyme, such as Mn superoxide dismutase (MnSOD), glutamine synthetase (GS), pyruvate carboxylase, and arginase. Mn‐based nanozymes have demonstrated various enzyme‐like activities.^[^ ^30^ ^]^ Here we synthesized a Mn‐based SAC (Mn‐N/C) that manifests peroxidase‐like activity, efficiently fostering the production of ROS. The impact of SACs on the tumor immune microenvironment remains largely unexplored. We reveal that Mn‐N/C incudes ICD in tumor cells by ROS and enhances the cross‐antigen presentation of DCs, consequently activating CD8^+^T anti‐tumor immunity. Moreover, we uncover that ROS‐triggered cytosolic DNA sensing by cGAS‐STING stimulates type I IFN signaling, which is critical for Mn‐N/C‐mediated enhancement of DC cross‐priming and subsequent CD8^+^T immune response. In fact, Mn^2+^ itself is a potent activator of cGAS, instigating the production of type I IFNs and bolstering anti‐tumor immunity.^[^ ^21^ ^]^ Following treatment with Mn‐N/C, we detected the presence of Mn^2+^ in the supernatant. The simultaneous release of cytosolic DNA and Mn^2+^ by Mn‐N/C appears to cooperatively activate the cGAS‐STING pathway. Moreover, we observed a superior therapeutic efficacy and more potent anti‐tumor immune response prompted by Mn‐N/C compared to MnCl_2_. Additionally, the synergy between Mn^2+^ and DNA in activating the cGAS‐STING‐IFN signaling cascade may contribute to the advantage of Mn‐based nanozyme over other elements‐based nanozyme. Of note, a PEGylated Mn‐based SAC has been reported to have significant anti‐tumor efficacy through the generation of ROS and photothermal activity.^[^ ^15a^ ^]^ Nevertheless, it remains a possibility that Mn‐N/C possesses photothermal conversion properties, which could potentially contribute to its anti‐tumor effects.

Type I IFNs which mainly include IFNα and IFNβ are important regulators of innate and adaptive immune responses, such as activation of DCs and promoting survival of CTLs.^[^ ^31^ ^]^ Our previous studies have demonstrated that type I IFN signaling is generally deactivated during tumor progression,^[^ ^23^, ^32^ ^]^ suggesting that re‐activating type I IFN signaling would be critical for anti‐tumor immunity. Type I IFNs can be induced following recognition of cytosolic DNA/RNA by host pattern recognition receptors (PRRs) like Toll‐like receptors (TLRs), cGAS, and retinoic acid‐inducible gene I (RIG‐I).^[^ ^33^ ^]^ It is reported that tumor‐derived DNA triggers the production of type I IFNs in CD11c^+^ tumor‐infiltrating DCs through cGAS‐STING pathway, thus priming the CD8^+^ anti‐tumor immune response.^[^ ^34^ ^]^ Our data indicate that the release of cytosolic DNA, including both mtDNA and gDNA triggered by ROS, stimulates the induction of type I IFNs in tumor cells via cGAS‐STING pathway. Of course, DNA released from dead cells may also elicit type I IFN production in immune cells like DCs or macrophages. Type I IFNs may exert their influence on various immune cells, including regulatory T cells (Tregs),^[^ ^35^ ^]^ and myeloid‐derived suppressor cells (MDSCs),^[^ ^36^ ^]^ potentially affecting their functional status. Other potential cellular targets of Mn‐N/C‐induced IFNs in TME and the involved effects may not be excluded.

In this study, we performed RNA sequencing and utilized IFNAR1 deficient mice to demonstrate the dependency of Mn‐N/C‐mediated anti‐tumor immune response on the activation of type I IFN signaling. Moreover, Mn‐N/C treatment induces PD‐L1 expression on tumor cells through activating type I IFN signaling, thereby potentiating the sensitization of PD‐L1 blockade. These findings provide a new clue for enhancing the efficacy of immunotherapy. Certainly, in addition to type I IFN signaling, we cannot rule out the potential involvement of alternative mechanisms underlying Mn‐N/C‐induced immune activation and its synergistic effect when combined with PD‐L1 blockade.

## 4 Conclusion

In summary, we constructed a manganese (Mn)‐based single‐atom nanozyme termed Mn‐N/C, demonstrating efficient peroxidase‐like activity. Mn‐N/C drives a substantial generation of ROS, subsequently inducing ICD in tumor cells and robustly potentiating CD8^+^T anti‐tumor immune response. We further uncover that Mn‐N/C treatment induces the concurrent release of cytosolic DNA and Mn^2+^, orchestrating a coordinated activation of the cGAS‐STING‐IFN signaling cascade. Moreover, type I IFN signaling is essential for Mn‐N/C‐mediated anti‐tumor immune response. Overall, our study offers novel insights poised to augment the efficacy of immunotherapy.

## 5 Experimental Section

### Synthesis and Characterization of Mn‐N/C

Zn(NO_3_)_2_·6H_2_O (1.069 g, 3.6 mmol) was dissolved in 15 mL methanol, into which Mn(acac)_3_ (0.077 g, 0.22 mmol) was added under stirring. After 5 min, 10 mL of methanol containing 2‐methylimidazole (1.161 g, 14.2 mmol) was added and kept under stirring for 5 min. Then, the mixed solution was kept at room temperature for 20 h without stirring. The Mn(acac)_3_@ZIF‐8 solid products were collected by centrifugation, washed four times with methanol, and dried overnight in a vacuum. The powder of Mn(acac)_3_@ZIF‐8 was placed in a tubular furnace and heated to 900 °C under argon gas (200 mL min^−1^) for 3 h at a heating rate of 5 °C min^−1^. After calcination, the samples were cooled to room temperature to obtain Mn‐N/C. Scanning electron microscopy (SEM), transmission electronic microscopy (TEM), energy dispersive spectrometry (EDS), X‐ray diffraction (XRD), and X‐ray photoelectron spectroscopy (XPS) were applied for further characterization.

### Synthesis of MnO_2_ Nanoparticles (NPs)

MnCl_2_ (0.39 g, 3 mm) and KMnO_4_ (0.316 g, 2 mm) were weighed separately and dissolved in distilled water (80 mL). Followed by stirring for 30 min, they were reacted in a reactor for 6 h (160 °C). After that, the reactor was naturally cooled to room temperature, and the MnO_2_ NPs were purified by centrifugation.

### Peroxidase (POD)‐Like Activity

Mn‐N/C (10 µL, 0.2 mg mL^−1^) and 3,3′,5,5′‐tetramethylbenzidine (TMB) (10 µL, 5 mm) were first added to NaAc‐HAc buffer (80 µL, 0.5 mm, pH 4.0) and incubated together for 10 min at 37 °C or the different temperatures ranged from 20 to 80 °C. Then, a given concentration of H_2_O_2_ (10 µL) was added into the solution and kept at 37 °C or the different temperatures ranged from 20 to 80 °C for 10 min. UV–vis spectra were conducted to record the fluctuation. To determine the activity of Mn‐N/C at different pH, various buffer solutions were used. For the pH between 3.6 and 5.6, the NaAc‐HAc buffer was used; For the pH between 6.0 and 7.6, the PB buffer was used; and for the pH between 8.0 and 10.0, the NH_3_‐NH_4_Cl buffer was used.

### MB Degradation by ROS

Mn‐N/C (10 µL, 0, 100, 200, 500, 1000, and 2000 µg mL^−1^) and MB solution (10 µL, 0.01%) were added into NaAc/HAc (pH 4.0, 70 µL). After that, H_2_O_2_ (10 µL, 3 m) was added into the solution and kept at 37 °C for 30 min. Finally, the supernatant was washed by centrifugation, and the absorbance of MB at 660 nm was tested.

### Detection of •OH by Electron Paramagnetic Resonance (EPR)

For EPR detection, 5,5‐Dimethyl‐1‐pyrroline N‐oxide (DMPO) was employed as the •OH trapping agent. Mn‐N/C (1 mg) was added into the NaAc‐HAc buffer (pH 4.0) containing H_2_O_2_ (1.0 mL) and 100 µm DMPO. The above mixture solutions were transferred to a quartz tube for EPR assay after mixing by sonication for 5 min.

### Density Functional Theory (DFT) Calculations

We used the DFT as implemented in the Vienna Ab initio simulation package (VASP) in all calculations. The exchange‐correlation potential was described by using the generalized gradient approximation of Perdew–Burke–Ernzerhof (GGA‐PBE). The projector augmented‐wave (PAW) method was employed to treat interactions between ion cores and valence electrons. The plane‐wave cutoff energy was fixed to 520 eV. Given structural models were relaxed until the Hellmann–Feynman forces were smaller than −0.02 eV Å^−1^ and the change in energy smaller than 10^−5^ eV was attained. The adsorption energy (Eads) was calculated as:

(1) $$E_{\mathrm{ads}}=E\left(\mathrm{system}\right)-E\left(\mathrm{catalyst}\right)-E\left(\mathrm{species}\right)$$

where *E*(system), *E*(catalyst), and *E*(species) were the total energy of the optimized system with adsorbed species, the isolated catalyst, and species, respectively. The Gibbs free energy change was defined as:

(2) ΔG=ΔE+ΔZPE−TΔS

where ΔE was the electronic energy calculated with VASP, ΔZPE, and ΔS were the zero‐point energy difference and the entropy change between the products and reactants, respectively, and *T* was the temperature (298.15 K).

### Cell Culture

MC38, MC38‐OVA, and CT26 mouse colon adenocarcinoma cell lines were kindly provided by Dr. Bin Ma (School of Biomedical Engineering and Medical‐X Research Institute at Shanghai Jiao Tong University). *Cgas^−/−^* MC38 and *Sting^−/−^* MC38 tumor cells were obtained from the laboratory of Dr. Liufu Deng (School of Pharmacy, Shanghai Jiao Tong University). MC38 and MC38‐OVA tumor cells were cultured in DMEM (Gibco), and CT26 cells were cultured in RPMI‐1640 (Gibco) at 37 °C under 5% CO_2_. Both of the culture media were supplemented with 10% fetal bovine serum (FBS, Gibco) and 100 U mL^−1^ penicillin/streptomycin (Gibco). Regular testing was performed to ensure free of mycoplasma contamination.

### Intracellular ROS Imaging

CT26 cells were seeded into 24‐well culture plates and cultured at 37 °C for 24 h. Then, cells were incubated with H_2_O_2_ for 12 h at a concertation of 100 µm. All wells were washed by PBS and then Mn‐N/C (100 µg mL^−1^) was added. After 4 h, the medium was replaced with fresh medium. After 24 h, the cells were washed and incubated with DCFH‐DA for 30 min and stained by Hoechst (1 µg mL^−1^) for another 9 min. Fluorescent imaging of DCFH‐DA was done with 488 nm excitation and 510–540 nm emission. Hoechst was excited with a 405 nm laser with emission collected at 420–460 nm.

### CRT Immunofluorescence Staining

CT26 cells were treated with H_2_O_2_, Mn‐N/C, or Mn‐N/C plus with H_2_O_2_ as described above. After 24 h, cells were gently washed 3 times with serum‐free DMEM, fixed with 4% paraformaldehyde for 15 min at room temperature, and then gently washed 3 times with PBS. The fixed cells were blocked with 200 µL of 5% BSA for 2 h. After that, cells were incubated with primary antibody anti‐CRT (1:100, Abclonal, cat#A1066) over night at 4 °C. After washing, cells were incubated with secondary antibody goat anti rabbit‐FIuor‐647 diluted with 1% BSA (1:1000) for 2 h. The cells were stained by Hoechst (1 µg mL^−1^) for another 9 min using a standard procedure. Finally, samples were imaged by Confocal laser scanning microscopy (CLSM). CRT was excited with a 651 nm laser with emission collected at 667 nm.

For CRT staining of tumors, tumor tissues were frozen in Tissue‐Tek O.C.T. compound and crysectioned into 6 µm sections using Leica CM3050 S Cryostats. The frozen sections were fixed with acetone at −20 °C, then washed with PBS two times. The slides were blocked with 5% goat serum with 1% BSA‐PBS for 1 h at room temperature. After that, the slides were incubated with primary antibody anti‐CRT (1:100) overnight at 4 °C. After washing, cells were incubated with secondary antibody goat anti‐rabbit Alexa Fluor 488 for 1 h at room temperature. After washing 3 times, the sections were mounted with ProLong Gold with DAPI. Fluorescent images were captured using a Leica DM2500 microscope.

### Mice

All mice used in these experiments were six‐ to eight‐week‐old and housed under specific pathogen‐free conditions in the animal facility at Ren Ji Hospital. WT C57BL/6J and BALB/c mice were purchased from Shanghai Slac Laboratory Animal Center. OT‐1 mice were purchased from Shanghai Model Organisms Center. *Ifnar1^−/−^* mice were generously provided by Dr. Chunsheng Dong (Institute of Biology and Medicine, Soochow University). The OT‐1 and *Ifnar1* ^−/−^ mouse colonies were maintained in the animal facility.

### Animal Tumor Models

For the transplanted colorectal tumor model, 1 × 106 MC38 or 2 × 106 MC38‐OVA in 100 µL PBS were subcutaneously injected into the right flank of WT C57BL/6J, or *Ifnar1^−/−^* mice. 1 × 106 CT26 in 100 µL PBS were subcutaneously injected into WT BALB/c mice. When the tumor size reached 60 mm^3^, mice were intratumorally injected with saline (50 µL), MnCl_2_ (5 mg kg^−1^, 50 µL), or Mn‐N/C (70 mg kg^−1^, 50 µL) every 3 days. The tumor volume and mice weight were monitored every 3 days. Tumor volumes were measured along the maximum and perpendicular diameters (a, b) and calculated using the formula V = a × b × b/2. Mice were euthanized, and the tumor tissues were collected until the tumor volume reached 800–1000 mm^3^.

For the bilateral colorectal tumor model, 1 × 106 MC38 tumor cells were subcutaneously injected into the right flank of WT C57BL/6J mice (Day −7). Four days later, 1 × 106 MC38 tumor cells were subcutaneously injected into the left flank of the same mouse (Day −3). Three days after the secondary injection, mice were intratumorally administered with saline (50 µL) or Mn‐N/C (70 mg kg^−1^, 50 µL) every 3 days. The next day after Mn‐N/C treatment, mice received intravenous injection of anti‐PD‐L1 antibody (1 mg kg^−1^, 100 µL) every 3 days. Tumor volumes were measured every 3 days. Mice were euthanized and the tumor tissues were collected until the volume of the first tumor reached 800–1000 mm^3^.

### In Vitro Treatments

When tumor cells reached 80−90% confluence, cells were exposed to culture medium containing Mn‐N/C (100 µg mL^−1^), H2O2 (100 µm), H2O2 (100 µm) plus with Mn‐N/C (100 µg mL^−1^) for 24 h. For DNA depletion, 10 U mL^−1^ of DNase (Sangon, cat#B618252) was added to the culture medium. For ROS scavenging, 1 mm N‐acetylcysteine (NAC) was used as an anti‐oxidant. Other inhibitors including 1 µm STING inhibitor H151 (TargetMol, cat#T5674), or 20 µg mL^−1^ anti‐IFNAR1 neutralizing antibody (BioXCell, cat#BE0241) were added together with Mn‐N/C and H_2_O_2_.

### Inductively Coupled Plasma Mass Spectrometry (ICP‐MS)

MC38 cells were treated with Mn‐N/C (100 µg mL^−1^) plus with H_2_O_2_ (100 µm) for 24 h. After that, the culture medium was collected and centrifugated at 12 000 rpm for 10 min to remove Mn‐N/C material and cellular debris. Subsequently, the supernatant was acquired and underwent assessment for manganese elemental content using an Agilent 7700 Inductively Coupled Plasma Mass Spectrometer.

### Flow Cytometry

To analyze the infiltration of immune cells in TME, tumors were finely minced and incubated in DMEM supplemented with 1 mg mL^−1^ collagenase D (Sigma, cat#C5138) and 100 µg mL^−1^ DNase I, and mechanically and enzymatically dissociated at 37 °C for 1 h. A complete medium with FBS was added to the mixture to terminate digestion, and the mixture was filtered with a 70 µm cell strainer, washed with PBS once, and resuspended with 1% BSA‐PBS containing 1 mm EDTA. For the analysis of DCs in the draining lymph nodes, the lymph nodes were gently squeezed and passed through a 70 µm cell strainer. After washing with PBS, the cells were resuspended in 1% BSA‐PBS containing 1 mm EDTA.

For the cell surface marker staining, the isolated cells were incubated on ice with anti‐mouse CD16/32 antibody (Biolegend, cat#101 302) for 15 min to block non‐specific binding of Fc receptors. The cells were then stained with antibodies anti‐CD45 APC/Cyanine7 (Biolegend, Clone30‐F11, cat#103 115), anti‐CD3‐FITC (Biolegend, Clone17A2, cat#100 203), anti‐CD3‐APC (Biolegend, Clone17A2, cat#100 206), anti‐CD4‐APC (Biolegend, CloneGK1.5, cat#100 411), anti‐CD8‐PE (Biolegend, CloneYTS156.7.7, cat#126 607), anti‐CD8‐PerCP/Cyanine5.5 (Biolegend, CloneYTS156.7.7, cat#126 609), anti‐PD‐L1‐PE (Biolegend, Clone10F.9G2, cat#124 307) antibody or anti‐CD45‐APC/Cyanine7 (Biolegend, Clone30‐F11, cat#103 115), anti‐CD11c‐Bv510 (Biolegend, CloneN418, cat#117 337), anti‐I‐1/I‐E‐Alexa Fluor 700 (Biolegend, CloneM5/114.15.2, cat#107 621), anti‐CD103‐PE/Cyanine7 (Biolegend, Clone2E7, cat#121 425), and anti‐CD80‐PE (Biolegend, Clone16‐10A1, cat#104 707), anti‐CD86‐Brilliant Violet 650 (Biolegend, CloneGL‐1, cat#105 035) antibodies on ice for 30 min. After washing with PBS, the cells were resuspended in FACS buffer containing DAPI (1:2000) and incubated for 10 min. Cell acquisition was performed using the LSR Fortessa flow cytometer (BD Biosciences), and the data were analyzed using Flowjo software (Tree Star).

For the intracellular staining of CD8^+^ T cells, a total of 1 × 106 cells were incubated with Cell Stimulation Cocktail (eBioscience, cat#00‐4970‐93) and BrefeldinA (eBioscience, cat#88‐8823‐88) for 6 h, and stained with anti‐CD45‐APC/Cyanine7 (Biolegend, Clone30‐F11, cat#103 115), anti‐CD3‐APC (Biolegend, Clone17A2, cat#100 206), and anti‐CD8‐PE (Biolegend, CloneYTS156.7.7, cat#126 607), or anti‐CD8‐PerCP/Cyanine5.5 (Biolegend, CloneYTS156.7.7, cat#126 609) antibodies for 30 min. After washing with PBS, the cells were fixed with fixation buffer (eBioscience, cat#88‐8823‐88) for 30 min. Cells were stained with anti‐IFN‐γ‐AF700 (Biolegend, Clone XMG1.2, cat#505 823), or anti‐IFN‐γ‐APC/cyanine5.5 (Biolegend, Clone XMG1.2, cat#505 849), anti‐Granzyme B‐Percp/Cyanine5.5 (Biolegend, Clone QA16A02, cat#372 211), anti‐TNFα‐PE (Biolegend, Clone MP6‐XT22, cat#506 305), anti‐Perforin‐Pacific Blue (Biolegend, Clone S16009A, cat#154 311) antibodies diluted in permeabilization buffer for 30 min. Cell acquisition was performed using the LSR Fortessa flow cytometer (BD Biosciences), and the data were analyzed using Flowjo software (Tree Star). The gating strategies for flow cytometry are shown in Figure S5 (Supporting Information).

### DCs Cross‐Priming Assay

CD103^+^ DCs (MHCII^hi^CD11c^+^CD103^+^) were sorted out from draining lymph nodes of MC38‐OVA tumor‐bearing mice after one‐time Mn‐N/C injection and co‐cultured with OT‐1 CD8^+^T cells at a ratio of 1:10 for 3 days. T cells were labeled with 2.5 mm CFSE, and the CFSE levels of CD8^+^T cells were analyzed by FACS. The cytotoxic function of CD8^+^T cells was assessed by intracellular staining of IFNγ, Granzyme B, and TNFα by FACS.

### ROS Detection

The tumor cells were exposed to a culture medium containing Mn‐N/C (100 µg mL^−1^), H2O2 (10 µm), H2O2 (10 µm) plus with Mn‐N/C (100 µg mL^−1^) for 24 h. CellROX (Beyotime, cat#S0033M) was added to the samples at a final concentration of 10 µm and incubated at 37 °C for 20 min. The samples were prevented from light and promptly analyzed by FACS using FITC excitation.

### H_2_O_2_ Detection

Freshly obtained tumor tissues were homogenized at a ratio of 100 µL of lysis solution per 20 mg of tissue. The homogenates were centrifuged at 4 °C for ≈12 000 g for 3–5 min. The supernatant was taken for subsequent measurement. H_2_O_2_ concentration was measured by using an H_2_O_2_ detection kit (Beyotime, cat#S0038).

### GSH Measurement

The glutathione (GSH) contents in cell and tumor tissues were determined using a GSH and GSSG (oxidized glutathione disulfide) assay kit (Beyotime, cat#S0053) following the manufacturer's instructions.

### Quantitative Reverse Transcription PCR

Total RNA was extracted from the samples using Trizol (TIANGEN) following the manufacturer's instructions. The extracted RNA was subjected to reverse transcription using HiScript III RT SuperMix (Vazyme, cat#R333‐01) to generate complementary DNA. Real‐time PCR was performed using SYBR Green Master Mix (Vazyme, cat#Q121‐02). The gene expression levels were determined by calculating the cycle threshold (Ct) set within the linear range of DNA amplification. The relative expression was calculated using the cycle threshold method (2^−ΔΔCt^), with normalization to a housekeeping gene (*gapdh*). The primer sequences used can be found in Table S1 (Supporting Information).

### Detection of Cytosolic DNA

Following a 24‐h treatment with vehicle or Mn‐N/C, tumor cells were subjected to the extraction and detection of total DNA and cytosolic DNA as described.^[^ ^37^ ^]^ In brief, the treated cells were harvested and split into two equal aliquots. One aliquot was used as a normalization control for total mtDNA and gDNA. The pellet was resuspended in a solution containing 500 µL of 50 mm NaOH and boiled for 30 min to dissolve the DNA. 50 µL of 1 m Tris‐HCl (pH 8.0) was added to neutralize the pH and then centrifuged at 12 000 rpm for 10 min to remove intact cells. The other aliquot was resuspended in a cytoplasmic extraction buffer containing 150 mm NaCl, 50 mm HEPES (pH 7.4), and 20 mg mL^−1^ digitonin (Sigma, cat#300 410). The suspension was gently incubated for 10 min to permeabilize the plasma membrane, followed by centrifugation at 980 g for 3 min × 3 times. Lastly, the cytosolic supernatants were transferred to fresh tubes and centrifuged at 9000 g (maximal speed of the centrifuge) for 10 min to remove any cellular debris, yielding the pure cytoplasmic supernatant. DNA from the two equal aliquots was isolated using the QuickPure DNA MiniPrep Kit (Vazyme, cat# DC112). Quantitative real‐time PCR was performed using gDNA primers (*Tert* and *Plog1*) and mtDNA primers (*Dloop1* and *Dloop2*). The Ct values obtained from the total cell extracts were used as the normalization controls.

### RNA Sequencing

The MC38 tumor‐bearing mice with vehicle or Mn‐N/C treatment were anesthetized. The tumor tissues were harvested and snap‐frizzed in liquid nitrogen. RNA from tumor tissues was extracted using Trizol. RNA sequencing was conducted by the Novel Bio Bio‐Pharm Technology Co., Ltd. Differentially expressed genes were identified with a log‐fold change>1.8 and false discovery rate (FDR)<0.05. Gene functional enrichment analysis was conducted using GSEA. The expression changes in mouse transcriptome data were evaluated using log_2_FC expression. The extent of enrichment and statistical significance were measured using a normalized enrichment score (NES) and false discovery rate (FDR), respectively. The heat map was generated through the analysis of normalized expression data by log‐transformation. The color mapping was set to a red‐green color scheme, where red indicates upregulation and green represents downregulation.

### Data Availability

RNA‐sequencing data that support the findings of this study have been deposited into the Gene Expression Omnibus (GEO) under accession code GSE249852.

### Statistical Analysis

All presented results represent at least three independent experiments. The number of samples (*n*) for each panel was described in detail. No statistical method was used to determine the sample size in advance. Data are presented as mean ± SEM. Statistical analysis was performed using GraphPad Prism 9.0 software. A two‐tailed unpaired Student's *t*‐test was used for comparisons between the two groups. Multiple comparisons were performed using one‐way analysis of variance (ANOVA) or two‐way ANOVA, followed by Sidak or Tukey's test. Repeated‐measures two‐way ANOVA (mixed model) followed by Sidak's multiple comparisons test was used for the analysis of the tumor growth curve. The Kaplan–Meier curves were used to depict the survival function from lifetime data for mice (a tumor size of ≈700 mm^3^ was set as the endpoint). The long‐rank test or Gehan–Breslow–Wilcoxon test was used to analyze the differences between the groups. Statistical significance was denoted with ^*^(*p* < 0.05), ^**^(*p* < 0.01), ^***^(*p* < 0.001), ^****^(*p* < 0.0001) in the figures.

### Study Approval

All the animal studies were approved by the Animal Care and Use Committee at Renji Hospital, Shanghai Jiao Tong University School of Medicine (RJ2023‐137A).

## Conflict of Interest

The authors declare no conflict of interest.

## Author Contributions

W.Q. and J.C. contributed equally to this work. J.G., Y.Y., and D.Y. conceived the study and designed the research. W.Q., J.C., H.Z., C.H., S.D., and H.L. performed the experiments. D.Y. provided critical expertise and resources. J.G. and Y.Y. interpreted data. J.G., Y.Y., and W.Q. wrote the manuscript. All the authors discussed and read the manuscript.

## Supporting information

Supporting Information

## Data Availability

The data that support the findings of this study are available from the corresponding author upon reasonable request.
