# Supplementary material for: A Single‐Atom Manganese Nanozyme Mn‐N/C Promotes Anti‐Tumor Immune Response via Eliciting Type I Interferon Signaling
Source: Adv Sci (Weinh). 2024 Feb 2;11(14):2305979. doi: 10.1002/advs.202305979 (PMC11005736; doi:10.1002/advs.202305979)
Supplement: Supplementary file 1 — Supporting Information [file ADVS-11-2305979-s001.pdf]

ADVANCED SCIENCE
Open Access

Supporting Information

for *Adv. Sci.*, DOI 10.1002/advs.202305979

A Single-Atom Manganese Nanozyme Mn-N/C Promotes Anti-Tumor Immune Response via Eliciting Type I Interferon Signaling

Wen Qiao, Jingqi Chen, Huayuan Zhou, Cegui Hu, Sumiya Dalangood, Hanjun Li, Dandan Yang, Yu Yang* and Jun Gui**

Figure S1

A

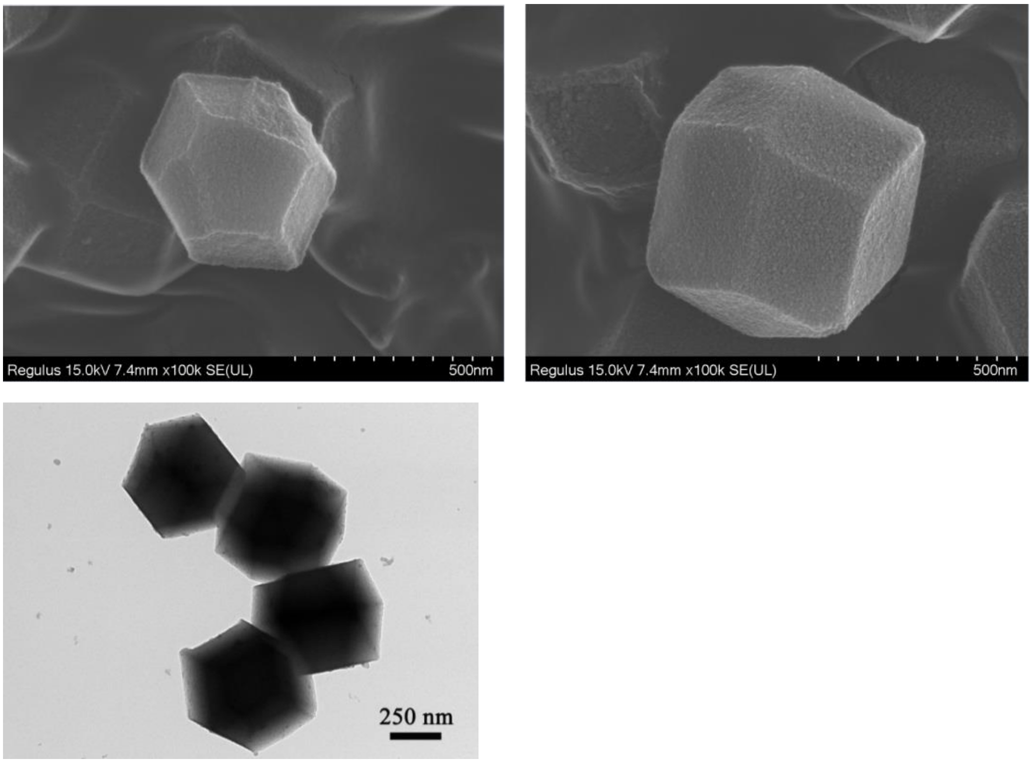

B

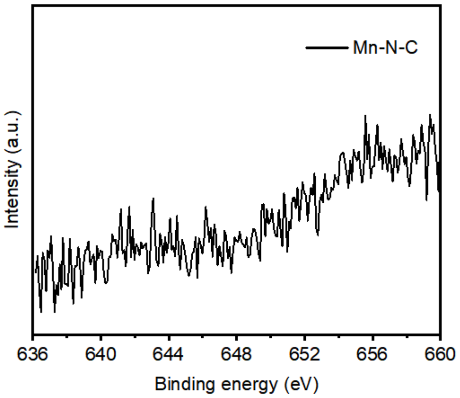

Figure S1. Characterization of single-atom manganese nanozyme Mn-N/C.

A. High-resolution SEM and TEM images of Mn-N/C.

B. XRD data of Mn-N/C, showing their amorphous feature.

Figure S2

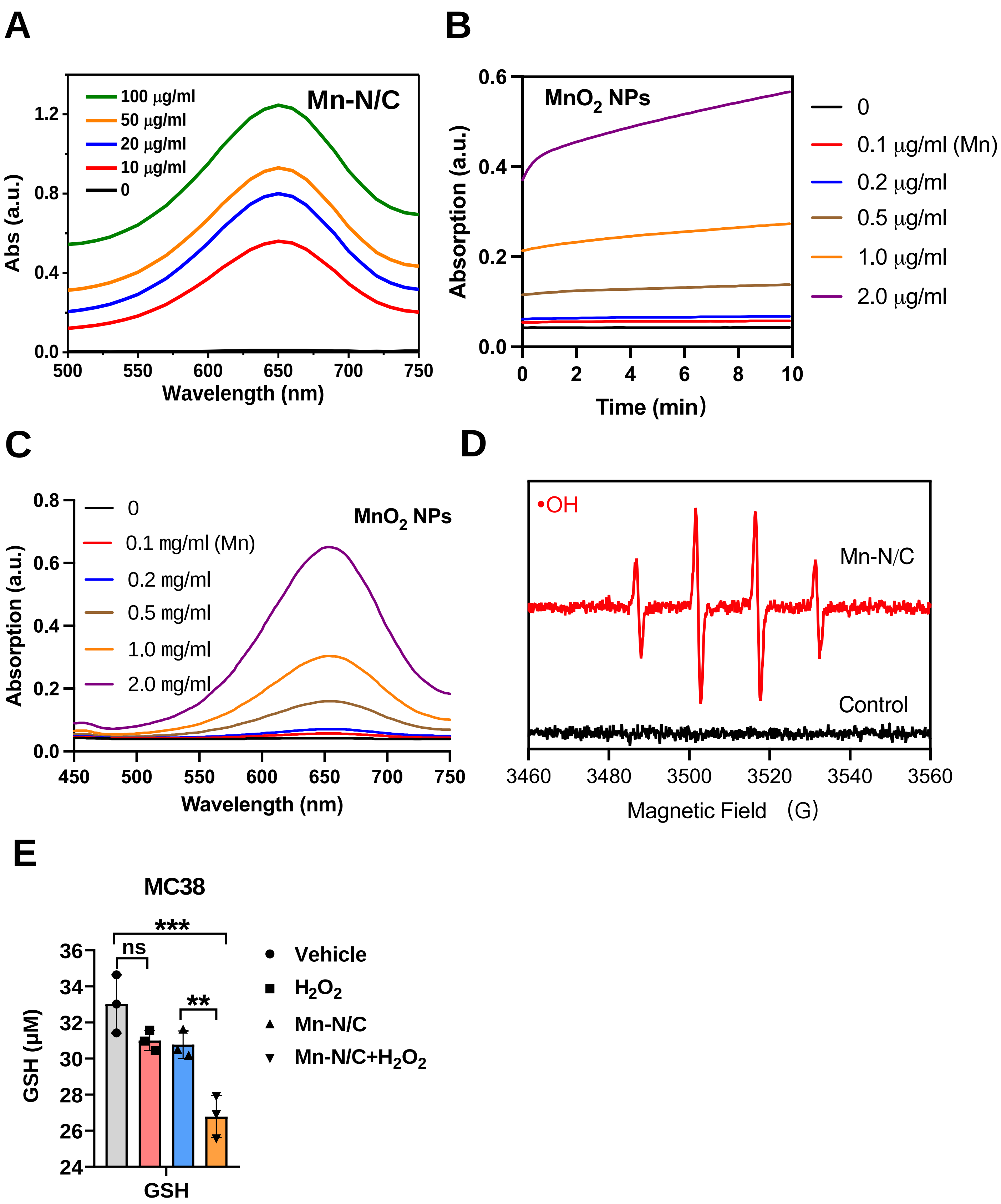
A
Mn-N/C
100 μg/ml
50 μg/ml
20 μg/ml
10 μg/ml
0
Abs (a.u.)
Wavelength (nm)
B
MnO2 NPs
0
0.1 μg/ml (Mn)
0.2 μg/ml
0.5 μg/ml
1.0 μg/ml
2.0 μg/ml
Absorption (a.u.)
Time (min)
C
MnO2 NPs
0
0.1 mg/ml (Mn)
0.2 mg/ml
0.5 mg/ml
1.0 mg/ml
2.0 mg/ml
Absorption (a.u.)
Wavelength (nm)
D
•OH
Mn-N/C
Control
Magnetic Field (G)
E
MC38
***
ns
**
Vehicle
H2O2
Mn-N/C
Mn-N/C+H2O2
GSH (μM)
GSH

Figure S2. The peroxidase-like activity of Mn-N/C and ROS generation.

A. The absorbance at different wavelengths in the absence (black) and presence of different concentrations of Mn-N/C.

B. The time-dependent absorbance changes of TMB (652 nm) in the absence (black) or presence of different concentrations of MnO_2.

C. The UV-Vis spectrum of TMB in the absence or presence of different concentrations of MnO_2.

D. EPR spectra for detection of •OH by DMPO.

E. GSH levels in MC38 tumors treated with H_2O_2 or Mn-N/C (100 μg/mL) for 24 hr. Data are shown as mean ± SEM (n=3). Two-tailed unpaired t-test was performed for the comparisons between two groups.

**, P<0.01; ***, P<0.001; ns, no significance.

Figure S3

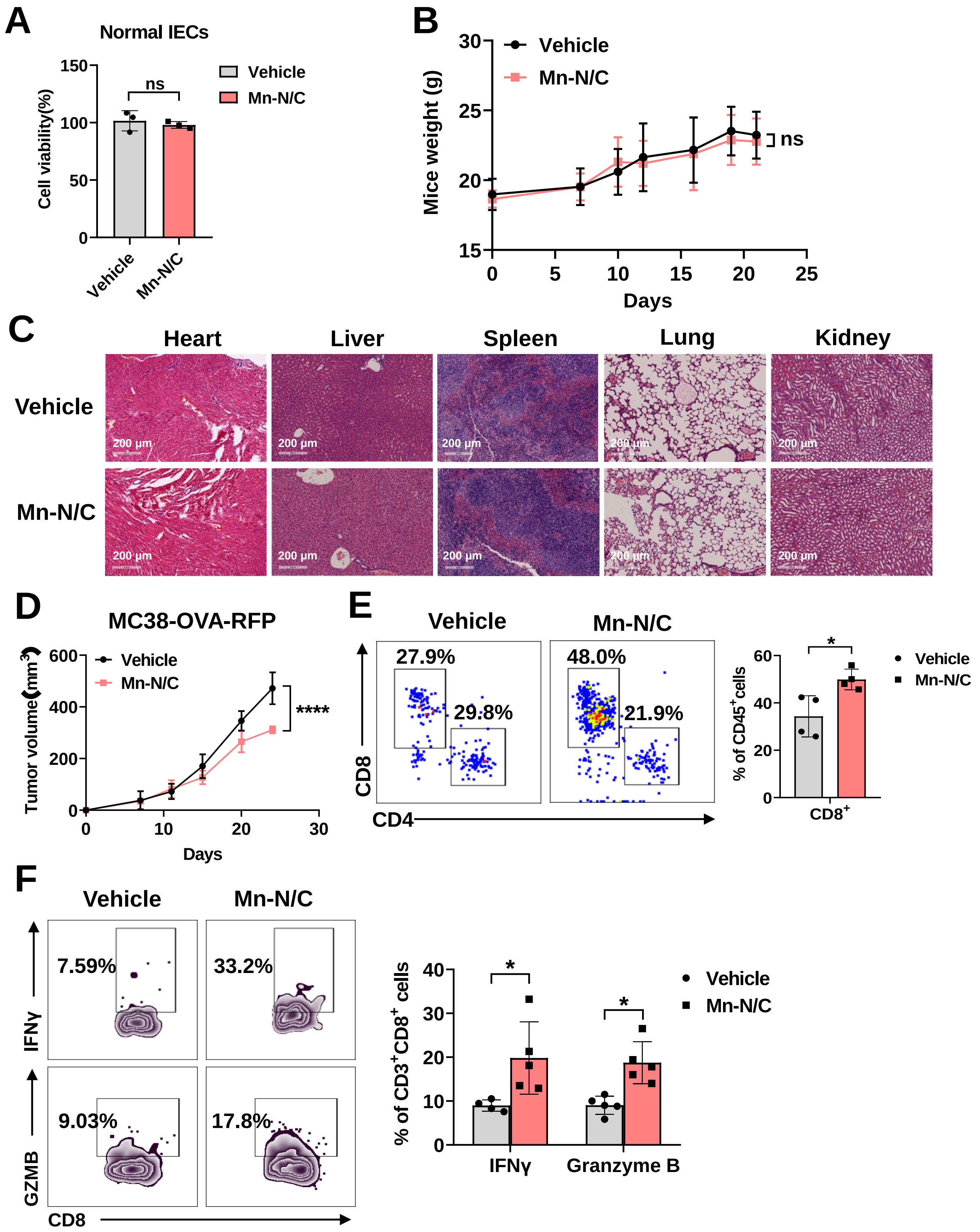
A
Normal IECs
Cell viability(%)
150
100
50
0
ns
Vehicle
Mn-N/C
B
Mice weight (g)
30
25
20
15
0
5
10
15
20
25
Days
ns
C
Heart
Liver
Spleen
Lung
Kidney
200 μm
D
MC38-OVA-RFP
Tumor volume (mm3)
600
400
200
0
10
20
30
****
E
27.9%
29.8%
48.0%
21.9%
CD8
CD4
% of CD45+cells
60
40
20
*
CD8+
F
7.59%
33.2%
9.03%
17.8%
IFNγ
GZMB
% of CD3+CD8+ cells
40
30
20
10
Granzyme B

Figure S3. Mn-N/C suppresses tumor growth without tissue damage.

A. The cell viability of normal intestinal epithelial cells (IECs) treated with vehicle or Mn-N/C for 24 hr by CCK8 assays. Quantitative data are shown as mean±SEM (n=3). Two-tailed unpaired t-test was performed for the comparison between two groups.

B. Body weight curve of CT26 tumor-bearing mice treated with vehicle or Mn-N/C. Data are shown as mean±SEM (n=4-5 mice per group). Two-way ANOVA (mixed model) and Sidak's multiple comparisons test were performed.

C. Representative IHC images of heart, liver, spleen, lung and kidney of CT26 tumor-bearing mice treated with vehicle or Mn-N/C.

D. Tumor growth curve of MC38-OVA-RFP in the WT mice treated with vehicle or Mn-N/C. Data are shown as mean±SEM (n=5 mice per group). Two-way ANOVA (mixed model) and Sidak's multiple comparisons test were performed.

E. FACS analysis (left) and quantification (right) of tumor-infiltrating $CD8^+$ T cells in the mice treated with vehicle or Mn-N/C as **D** described. Data are shown as mean±SEM (n=4 mice per group). Two-tailed unpaired t-test was performed for the comparisons between two groups.

F. FACS analysis (left) and quantification (right) of $IFN\gamma^+$ and Granzyme B^+ of tumor-infiltrating $CD8^+$ T cells in the mice treated with vehicle or Mn-N/C as **D** described. Data are shown as mean±SEM (n=4-5 mice per group). Two-way ANOVA and Sidak's multiple comparisons test were performed.

*, P<0.05; ****, P<0.0001; ns, no significance.

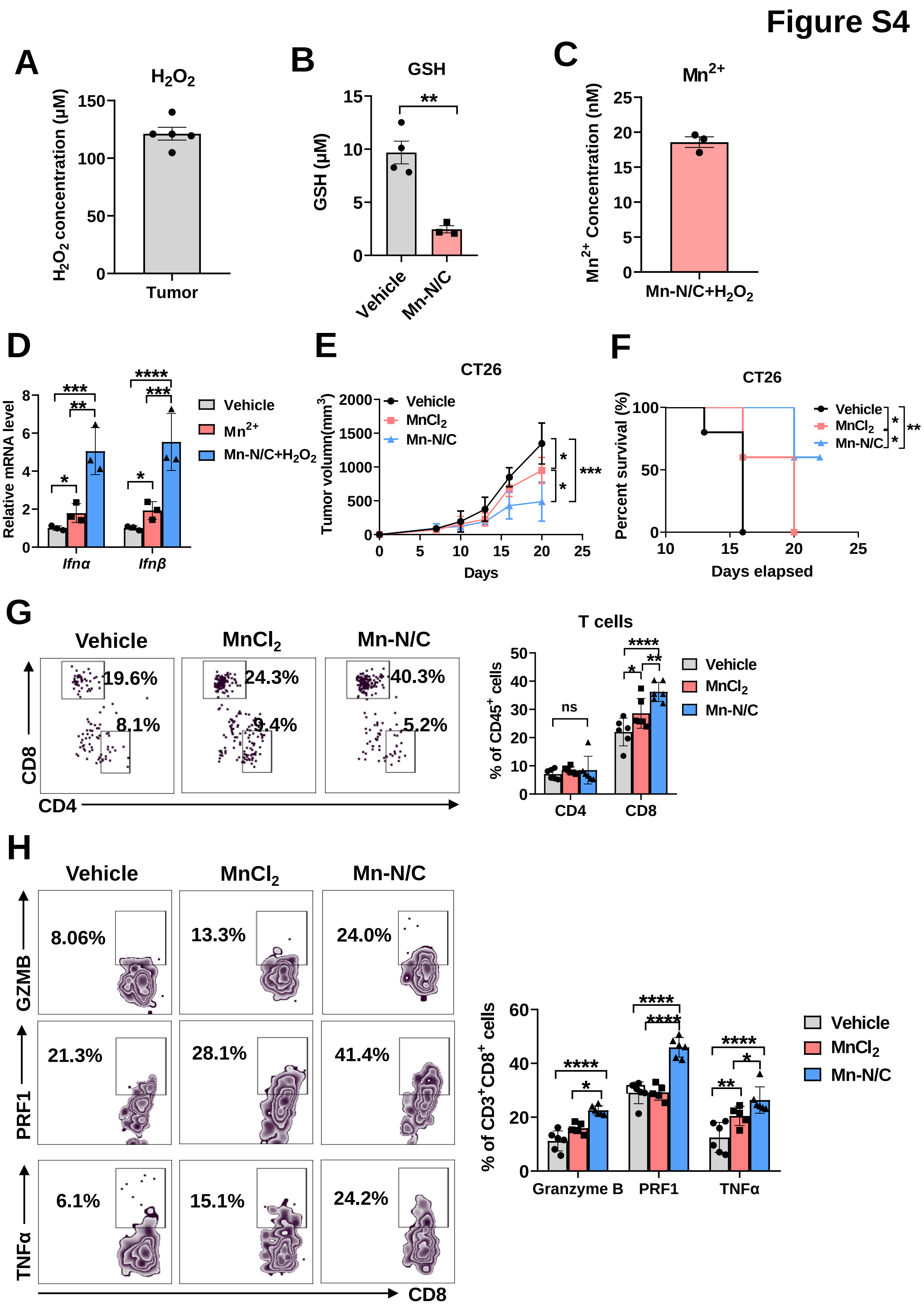

Figure S4
A
H_2O_2
H_2O_2 concentration (μM)
Tumor
B
GSH
GSH (μM)
**
Vehicle
Mn-N/C
C
Mn^{2+}
Mn^{2+} Concentration (nM)
Mn-N/C+H_2O_2
D
Relative mRNA level
Ifnα
Ifnβ
Vehicle
Mn^{2+}
Mn-N/C+H_2O_2
E
CT26
Tumor volumn(mm^3)
Days
Vehicle
$MnCl_2$
Mn-N/C
F
CT26
Percent survival (%)
Days elapsed
G
Vehicle
$MnCl_2$
Mn-N/C
19.6%
8.1%
24.3%
9.4%
40.3%
5.2%
CD8
CD4
T cells
% of CD45$^+$ cells
ns
H
Vehicle
$MnCl_2$
Mn-N/C
GZMB
8.06%
13.3%
24.0%
PRF1
21.3%
28.1%
41.4%
TNFα
6.1%
15.1%
24.2%
CD8
% of CD3$^+$CD8$^+$ cells
Granzyme B
PRF1
TNFα

Figure S4. Mn-N/C displayed superior tumor suppression compared to $MnCl_2$.

A. H_2O_2 concentration in CT26 tumor tissues. Data are shown as mean±SEM (n= 5 mice).

B. GSH levels in CT26 tumor tissue following vehicle or Mn-N/C treatment. Data are shown as mean±SEM (n=3-4 mice per group). Two-tailed unpaired t-test was performed for the comparisons between two groups.

C. Mn^{2+} concentration in the supernatant of MC38 tumor cells treated with Mn-N/C and H_2O_2 for 24 hr. After treatment, supernatants were subjected to high-speed centrifugation to remove residue Mn-N/C. The content of dissociated Mn^{2+} in the supernatant was determined by ICP-MS. Data are shown as mean±SEM (n=3).

D. Real-time PCR analysis of *Ifnα* and *Ifnβ* gene expression levels in MC38 tumor cells treated with Mn^{2+} (20 nM) or Mn-N/C combined with H_2O_2 for 24 hr. Data are shown as mean±SEM (n=3). Two-way ANOVA and Sidak's multiple comparisons test were performed.

E. Tumor growth curve of CT26 in the WT mice intratumorally injected with vehicle, $MnCl_2$ or Mn-N/C. Data are shown as mean±SEM (n=5 mice per group). Two-way ANOVA (mixed model) and Sidak's multiple comparisons test were performed.

F. Kaplan–Meier analysis of mice survival after vehicle, $MnCl_2$ or Mn-N/C treatment as described in **E** (n=5 mice per group).

G. FACS analysis (left) and quantification (right) of tumor-infiltrating $CD4^+$ and $CD8^+$ T cells in the mice treated with vehicle, $MnCl_2$, or Mn-N/C. Data are shown as mean±SEM (n=6 mice per group). Two-way ANOVA and Sidak's multiple comparisons test were performed.

H. FACS analysis (left) and quantification (right) of Granzyme B^+, $PRF1^+$, $TNF\alpha^+$ of tumor-infiltrating $CD8^+$ T cells in the mice treated with vehicle, $MnCl_2$, or Mn-N/C. Data are shown as mean±SEM (n=6 mice per group). Two-way ANOVA and Sidak's multiple comparisons test were performed.

*, $P<0.05$; **, $P<0.01$; ***, $P<0.001$; ****, $P<0.0001$

Figure S5

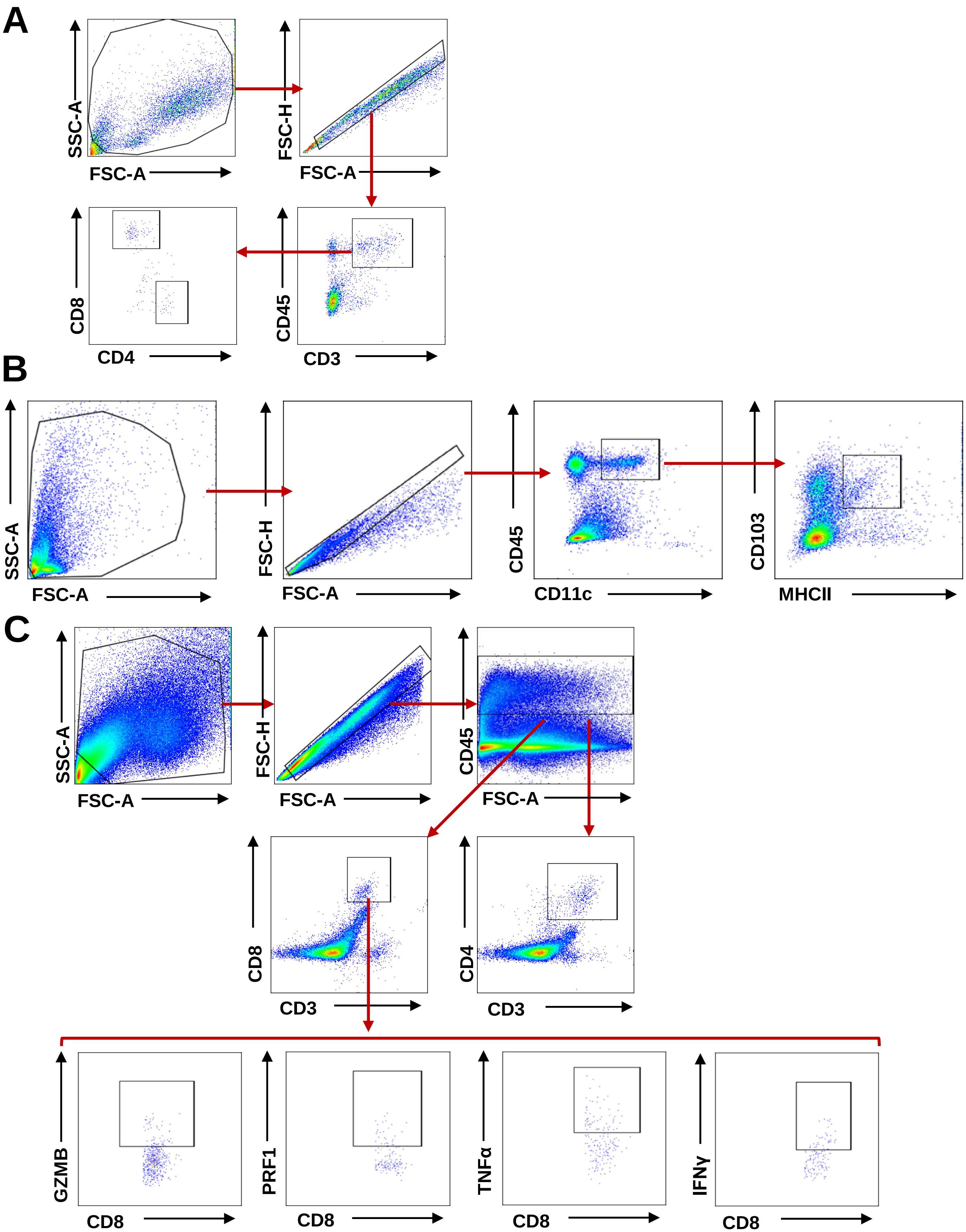

Figure S5. The gating strategies for flow cytometry.

A. Gating strategy for tumor-infiltrating $CD4^+$ and $CD8^+$T lymphocytes.

B. Gating strategy for DCs.

C. Gating strategy for cytotoxic molecules of tumor-infiltrating $CD8^+$T cells.

Table S1

Primers used in RT-PCR

**Primer name**	Sequence (5'-3')
Murine *Gapdh*-F	AGCTTGTCATCAACGGGAAG
Murine *Gapdh*-R	TTTGATGTTAGTGGGGTCTCG
Murine *Ifna*-F	ATTTCCCCTGACCCAGGAAGATG
Murine *Ifna*-R	CCCAGCACATTGGCAGAGG
Murine *Ifnb*-F	AGCTCCAAGAAAGGACGAACA
Murine *Ifnb*-R	GCCCTGTAGGTGAGGTTGAT
Murine *Cxcl10*-F	CCAAGTGCTGCCGTCATTTTC
Murine *Cxcl10*-R	GGCTCGCAGGGATGATTTCAA
Murine *Hmgb1*-F	GGCGAGCATCCTGGCTTATC
Murine *Hmgb1*-R	GGCTGCTTGTCATCTGCTG
Murine *Ddit3*-F	CTGGAAGCCTGGTATGAGGAT
Murine *Ddit3*-R	CAGGGTCAAGAGTAGTGAAGGT
Murine *Irf7*-F	TACCATCTACCTGGGCTTCG
Murine *Irf7*-R	GCTCCATAAGGAAGCACTCG
Murine *Pdl1*-F	GCTCCAAAGGACTTGTACGTG
Murine *Pdl1*-R	TGATCTGAAGGGCAGCATTTC
Murine *Isg15*-F	GGAACGAAAGGGGCCACAGCA
Murine *Isg15*-R	CCTCCATGGGCCTTCCCTCGA
Murine gDNA *Tert*-F	CTAGCTCATGTGTCAAGACCCTCTT
Murine gDNA *Tert*-R	GCCAGCACGTTTCTCTCGTT
Murine gDNA *Polg1*-F	GATGAATGGGCCTACCTTGA
Murine gDNA *Polg1*-R	TGGGGTCCTGTTTCTACAGC
Murine mtDNA *Dloop 1*-F	CCCTTCCCCATTTGGTCT
Murine mtDNA *Dloop 1*-R	TGGTTTCACGGAGGATGG
Murine mtDNA *Dloop 2*-F	CCCTTCCCCATTTGGTCT
Murine mtDNA *Dloop 2*-R	TGGTTTCACGGAGGATGG
